# Spirit of the Foreign Periodicals, &c.

**Published:** 1837-10-01

**Authors:** 


### Spirit of the Foreign Periodicals, &c.

### Memoir on some remarkable Cases of Organic Anomalies.

In the course of my studies, says Dr. Petrequin, in normal and pathological anatomy, I have had frequent occasion to meet with some organic anomalies of structure or arrangement, which are worthy perhaps of being communicated to the profession. Such irregularities of formation have, until the last few years, been either quite overlooked, or misunderstood; and hence we can draw little useful information on this very curious subject of enquiry, previous to the publication of the writings of Meckel, St. Hilaire, and Serres.* Numerous are the opportunities which occur in almost every dissecting-room to collect and to examine numerous varieties of irregular development; and it is to be sincerely regretted that so little use has hitherto been made of these. We have only to call to mind what unexpected light the study of monstrosities, in connexion with that of comparative anatomy, has thrown on the general laws of organo-genesy, to induce us to pay more attention to this subject. It is in this department of science, more perhaps than in any other one, that we recognise the truth of the saying, "exceptio probat regulam;" the very deviation from normal development and formation confirming, in the most beautiful and convincing manner, the great laws of philosophical anatomy.

Prompted by these motives we have collected a few instances of organic anomalies, which we shall now briefly describe.

* The subject of Philosophical anatomy was examined at great length in the last number of this Review. We therefore refer our readers to the article there published for an account of the history, as well as of the present state of the science.

*Urinary Apparatus.*

Case 1.—*Right Kidney provided with two Ureters; Left Kidney bilobular, and having two Pelves and two Ureters.*

A man. 55 years of age, was brought in a dying state to the Hôtel Dieu at Lyons.

On dissection, the prostate gland was found in an inflamed and suppurating condition; the mucous coat of the bladder was much thickened and corrugated. The right kidney was larger than its fellow. It was provided with two ureters, one of which arose, as usual, from the pelvis, and was very large and wide for about two inches in length, and then regained its ordinary dimensions. The second ureter arose from the lower part of the kidney; it was smaller than the other one, from which at first it was separated by about an inch, gradually however approaching near to it, in their course downwards.

The left kidney presented a singular arrangement of its parts. Instead of one large pelvis, there were two. The upper one, which was larger than the other, was imperfectly divided into several compartments; it terminated in an infundibulum, from which there was continued a ureter. The second, or lower pelvis also gave origin to a separate ureter. The two ureters gradually converged towards each other as they descended; and, for the lower two thirds of their course, they were firmly joined together. They opened, however, by distinct orifices into the bladder, The structure of this left kidney presented a curious appearance. It seemed as if there were two kidneys, united intimately the one with the other, and each of which was provided with its separate pelvis and ureter. Now this incomplete union realizes, in some respects, the fœtal condition of the organ, at an early period of intra-uterine existence.

Meckel has remarked that congenital malformations of the kidney are more frequently met with on the left than on the right side—a remark which is partially confirmed by the present case. I may add that the lobular structure of the kidney, which in man is deemed a monstrosity, is the normal condition of the organ in most of the lower mammiferous animals; most strikingly in the cetacea, in which the lobules are so numerous and so distinct from each other, that the viscus has often been compared to a cluster of grapes. It may be worthy of notice here, that most of the examples of what have been described as supernumerary or double kidneys are in truth only instances where one of the lobules has remained permanently detached from the others; and that in such cases, therefore, there is not a multiplication, but only a fissure, of the organ.

As to the ureters, their duplicity may be explained by the non-reunion of the roots, from which these tubes are primarily formed.

*Case* 2. In a woman, who died of phthisis pulmonalis in the wards of La Charité, the right kidney was found in a state of *hydronephrosis* or dropsy. From the distended pelvis arose the ureter, double at its origin and for four inches of its course, then becoming single from this point unto its insertion into the bladder. There was no anomaly on the left side.

In addition to the numerous instances of this sort which have been recorded, M. Lauth has described in the Archives de Medecine for 1834, a case, in which he found two ureters, the abnormal one of which was larger than the other and arose from the upper part of the kidney. M. Lediberder also, in the same Journal for 1835, has communicated two very interesting examples of a similar anomaly.

M. Isodore Geoffoy St. Hilaire has, in his admirable work on Teratology, laid down the position, that "an organ, having its analogue or fellow on the median line of the body, very rarely deviates from the normal formation." There are however very numerous exceptions to this law; and indeed it may even be doubted whether it can be admitted as such in philosophical anatomy.

*Case* 3. *Senile Hydro-nephrosis*; *Atrophy of two-thirds of the Cortical Substance; Fatty Transformation of*

*several of the mamillæ, and atrophy of the others.*

An octogenarian died in La Charité, at Lyons, in 1836. Dr. Petrequin found on dissection that the left kidney was in a dropsical state; all its mamillated substance had disappeared, with the exception of five of the mamillæ, which had become converted into a fatty-looking matter. The cortical substance, reduced to one-third of its usual proportion, formed as it were a sac or investment around the distended pelvis. Into this sac, which was filled with a limpid inodorous fluid, the remains of the five calices terminated. The ureter, of its ordinary dimensions, gave passage to this fluid, when the kidney was compressed with the hand. The right kidney was nearly sound in all its parts. At a few points only did it present traces of incipient fatty degeneration.

*Persistance of the Cavity of the Urachus.*

The function or use of this cord, which extends from the fundus of the bladder to the umbilicus, is still a subject of doubt to anatomists. The ancients supposed that it served as an excretory canal for the urine in the fœtus. But Cruveilhier, in his Anatomie Descriptive, assures us that he has never found it pervious even in the youngest fœtus. M. St. Hilaire, however, has collected the accounts of several cases, in which this organ was permeable. "When," says he, in his work on monstrosities, "its cavity is preserved from the bladder to the umbilicus, there is no outward sign of such a condition, provided the urinary passages remain free." This statement is not, however, strictly true in all cases. Hofmeister mentions that he has seen the urachus remain open after birth, in a child two years of age, the urethra being quite pervious all the time.* Perhaps also the following case may be regarded as another example of the same anomaly.

*Case* 4. Dr. Petrequin attended a lady, in whom there was an occasional oozing of a serous fluid from the umbilicus. No distinct orifice could be distinguished among the small folds there. As she advanced in years, the discharge became more frequent and more copious. The question comes to be, whence did this discharge proceed? Did it come from the bladder? or was it a secretion from a persistantly open urachus?

*Generative Apparatus. Persistance of the Testicles within the Abdomen.*

Dr. Petrequin informs us that he has met with three cases of this anomaly.

A man, 36 years of age, unmarried, was brought to the Hôtel Dieu at Lyons, in the last stage of acute meningitis. On dissection, the right testicle was found quite within the inguinal aperture, firmly attached to the peritoneum by its convex side, while the epididymis and the vas deferens had partially inserted themselves into the canal. The peritoneum had formed a fold, like the finger of a glove, and this passed some way down along the canal.

On the left side, two fingers could be readily passed from the cavity of the abdomen along the inguinal canal. The testicle, resting on the pubis, had advanced forwards to the two pillars or columnæ of the inner aperture, while the vas deferens and its accompanying blood vessels had protruded somewhat along the canal, and thus formed a slight hernia.

* According to the description given by Hofmeister, there existed at the umbilicus a fleshy, fungous, red, and pediculated excrescence, from which urine oozed out when it was pulled backwards. The urine flowed freely by the natural passage of the urethra. The excrescence was tied with a ligature, and fell off in the course of a few days. The umbilicus gradually cicatrised, and was covered with skin; and then all oozing ceased.

In the Foreign Periscope of a recent number of this Journal, there is a short account of a case, in which a fluid, somewhat resembling urine, oozed from the umbilicus.—*Rev.*

The two vasa deferentia, having a shorter *trajet* than usual, were twisted into numerous folds.

The anatomy of such a malformation, as the present, illustrates very beautifully the manner in which congenital hernia is produced; and at the same time affords a most interesting example of the persistence of one of the normal structures of embryotic life.

It may be here remarked, that the absence of the testicles in the scrotum cannot in any case be adduced as a proof of impotence of the individual. The medical jurist is, as a matter of course, aware of their occasional non-descent from the cavity of the abdomen, within which they are developed in all animals.

M. Mayor, of Lausanne, has published, in the Gazette Medicale for 1836, the account of a curious case, where the right testicle began to descend at 36 years of age.

*Existence of Three Mammæ in a Man, all of whose Children had the same Malformation.*

The interesting memoir of M. Bedor (Gazette Medicale, 1836) on Gynecomastie, has recalled to my memory, says Dr. Petrequin, a curious example of the above-mentioned anomaly.

*Case* 6. A middle-aged man had three distinct mammæ. Two were on the left side, and one of these, the supernumerary, was situated immediately above the other. He had five children, three sons and two daughters. The three boys were provided each with three mammæ, like their father; but only with this difference, that the supernumerary one was on the right side of the chest. The two daughters also were *trimammal;* in them the third mamma was on the left side, as in the case of their father.

Two of the daughters had married, and were the mothers of several children; but in these there was no trace of any anomaly of the mammæ.

The preceding case is very curious, as affording an example of the hereditary transmission, for one generation at least, of a structural irregularity.

*Existence of Four Mammæ in a Woman, the Supernumerary Ones situated in the Axilla.*

*Case* 7. In 1803, a woman, in the eighth month of pregnancy, consulted Dr. Martin, of Lyons, for two tumors, one in each axilla, immediately on the outer edge of the great pectoral muscles. They were of the size and shape of pullets' eggs, and seemed to consist entirely of skin and subjacent cellular tissue. On compressing them between the fingers, a yellowish milky-looking fluid was observed to ooze from their surface, similar to that which is frequently seen to come from the nipples of a pregnant woman. After being well squeezed in this manner, the tumors became smaller, and, as it were, skrunk from being emptied of their contents. From time to time they became painful and swollen; and this state never subsided, unless there was a discharge of serosity from them.

The regular mammæ were smaller than usual. After delivery, however, they became plump and distended, and during the whole period of lactation it was observed that when the child was applied to either of the regular mammæ, the supernumerary ones oozed out "un veritable lait."

A case somewhat similar to the one now recorded is related in the Dictionnaire des Sciences Medicales, tome xxx. by M. Champion de Bar-le-duc.

The anomaly, therefore, of supernumerary mammæ is of very rare occurrence. When there are four mammæ they are usually arranged in four symmetrical rows; thus presenting an arrangement somewhat analogous to what we find in the lower mammiferous animals. M. St. Hilaire, in the work to which we have already made more than one reference, makes the remark that the supernumerary mammæ, when they are situated laterally, afford milk; but not so, when they are situated in the mesian line. This is a very interesting remark, if it will be found to be strictly true, as we find that in all the lower animals (with the exception perhaps of some marsupialia) the teats are uniformly lateral, and never situated in the mesian line of the body.

On the whole, the anomalies " par exces" are much more rare than those " par defaut ;" and, in reference to the former, it may be laid down as a general truth that the organs, which are most frequently the seat of numerical anomalies, are precisely those of which the number is most variable in the different classes of animals. Here, therefore, we perceive the approximation, as it were of congenital malformations in the human subject, to the normal structure of animals, lower in the scale of organization.

We shall close this rather rambling article by describing a very curious case of

*Lateral Transposition of the Thoracic and Abdominal Viscera.*

A man, 20 years of age, was admitted into the Hôtel Dieu at Lyons, with a white-swelling of the knee. He refused to submit to amputation of the limb, and died a lingering death of marasmus.

On examining the body after death, the heart was found with its apex directed towards the false ribs of the right side. The aorta ascended from the left, and formed an arch backwards and towards the right, and then curving behind the root of the right lung passed along the right side of the dorsal vertebræ. From its arch there arose three great trunks; 1st, the arteria innominata, which divided into the left subclavian and the left carotid; 2dly, the right carotid; and 3rdly, the right subclavian. The vena cava superior descended along the left side of the spinal column as low down as the point of its bifurcation.

The left lung was found divided into three lobes, while the right one had only two.

The œsophagus pressed down on the right side of the mesian line, pierced the diaphragm in this direction, and joined the stomach on the right side of the epigastrium.

The liver occupied the left hypochondriac and part of the epigastric region.

The spleen lay in the hollow of the right hypochondrium.

The stomach was directed from the right to the left side; its large extremity occupying the right hypochondrium, and the pylorus resting on the left side under the concave surface of the liver.

The duodenum was directed from left to right, while the cæcum was situated in the left iliac fossa. The tissue of all the viscera was perfectly normal. During the life of this individual there had not been at any time observed any outward peculiarity of appearance, to indicate an irregularity of visceral arrangement.

The study of such an interesting case of transposition or ectopia is useful to the practical physician and surgeon, as well as to the physiologist.

If we attempt to explain the proximate cause, or mode of production of such an anomaly, we shall perhaps be led to agree with the hypothesis of M. Serres, that the development of the various organs of the body is primarily influenced by, and indeed dependant upon, the arrangement of their vascular trunks. According to this view, the " fons et origo mali" was the irregularity or transposition of the heart and blood-vessels.

It is of very rare occurrence that we ever find a partial transposition of the viscera; as for example the heart situated on the right side, or the liver on the left, while the rest of the viscera occupy their normal places. In general the displacement of one important organ *necessite* the displacement of other organs, with which it is more or less intimately associated in functions or connexion. But at present we cannot pursue this subject any further. To those who wish to make themselves acquainted with the histories of the most interesting cases on record, as well as the hypotheses which have been framed to explain their occurrence, we again recommend to their attentive perusal the work of M. St. Hilaire on Monstrosities.—*Gazette Medicale, April* 1837.

### Psychological Physiology.

### The Relation of the Weight of the Cerebral Mass with the Development of Intelligence.

Dr. Lelut informs us that he is about to publish a work, to be entitled "Nouveaux Rapports du Physique et du Moral," in which he proposes to illustrate, at considerable length, many very curious topics connected with the development of the brain, in reference to the mental and moral characters of individuals. Whether he is, or is not a phrenologist, we are not informed; but this matters little, provided he can satisfy the public that his data and facts are accurate and trustworthy.

Omitting some prefatory remarks, which are unimportant, we shall now lay before our readers the substance of his paper, which is inserted in one of the late numbers of the Gazette Medicale de Paris. The average weight of the encephalon (including the cerebrum, cerebellum, and tuber annulare or rachidian bulb), after the meninges have been stripped off, is in the healthy adult male about 1346 grammes, or three pounds and a half,* of which the cerebrum weighs 1170, and the cerebellum 176 grammes. In the female the weight of the encephalon is about 1-13th less than in the male. The following are the measurements of the encephalon in certain individuals, "dont la triste celebrité a jeté quelque jour sur leur psychologie."

1. Lhuissier, who murdered his mistress, and then cut the body up and threw the pieces into the Seine. Intelligence below the ordinary standard, and not cultivated—45 years of age—middle stature.

The encephalon weighed 1496 gram.
cerebrum . . . 1305
cerebellum . . 191

2. Belard, murderer of one of his relations; a tradesman in the Temple. Intelligence below the ordinary standard, and not educated—29 years of age—middle stature.

The encephalon weighed 1290 gram.
cerebrum . . . 1130
cerebellum . . 160

3. Bardon, an accomplice in a murder. Intelligence ordinary, and not cultivated—39 years of age—midd: stature.

The encephalon weighed 1384 gram.
cerebrum . . . 1204
cerebellum . . 180

4. Chandelet, the assassin of his uncle; a porter in the Rue de Charonne. Reason lively and exalted; propensities brutal and licentious—31 years of age—stature short.

The encephalon weighed 1192 gram.
cerebrum . . . 1010
cerebellum . . 182

5. Avril, the accomplice of the notorious Lacenaire. Intelligence ordinary, and not cultivated—27 years of age—middle stature.

The encephalon weighed 1310 gram.
cerebrum . . . 1130
cerebellum . . 180

6. David, murderer of his sister-in-law, who was the wife of a servant at the Hôtel des Invalides. Intelligence ordinary, but somewhat eccentric, and partially cultivated—34 years of age—stature rather tall.

The encephalon weighed 1420 gram.

N.B. It has been preserved in spirits, and the weight of the cerebrum and cerebellum, separately, cannot be stated.

7. Fieschi. Intelligence ordinary, but not cultivated, but active and proud—46 years of age—middle stature.

The encephalon weighed 1365 gram.
cerebrum . . . 1200
cerebellum . . 165

8. Guerin, accomplice of Chandelet (No. 4). Reason rather acute, developed, and somewhat educated—42 years of age—stature rather tall.

The encephalon weighed 1384 gram.
cerebrum . . . 1240
cerebellum . . 145

9. Lemoine, the assassin of the chamber-maid of Madame Dupuytren. Intelligence developed and cultivated—40 years of age—middle stature.

The encephalon weighed 1310 gram.
cerebrum . . . 1140
cerebellum . . 170

* An English pound of avoirdupois weight is equivalent to 423 grammes.

10. Lacenaire. Intelligence developed and cultivated—34 years of age—stature short.

The encephalon weighed 1355 gram.
cerebrum . . . 1205
cerebellum . . 150

*Remarks.* If we cast our eye over the preceding ten measurements, it will be seen that the medium or average weight may be stated, as we have said at first, at about 1340 or 1350 grammes. The heaviest on the list is that of Lhiussier (No. 1), and the lightest is that of Chandelet (No. 4); the former weighing 1496, and the latter only 1192 grammes.

If we endeavoured to establish any relation between the mental development or character of these criminals, and the weight of their encephala and of its two great divisions, it might be remarked that Bardon, Lhuissier and David, in whom the encephalon or the cerebrum was the most heavy, were not the most intelligent; that in Lacenaire, on the other hand, the brain weighed considerably less than in them, and that if in Chandelet, with his brutal passions, the cerebellum was large, the cerebrum weighed as low as it does in many idiots, and yet he indicated no want of intelligence.

But we have no intention to discuss this most difficult question, as we most willingly admit that a number of considerations ought to be taken into account, in such an enquiry. It might, for example, be objected that we have not sufficiently attended to the size and weight of the whole body in reference to the weight of the encephalic mass; nor to the relative weights of its two great parts, the cerebrum and the cerebellum; nor to the education of the individuals; and, even if we were provided with accurate data on these topics, phrenology might then step in, and remind us that we have forgotten to attend to the comparative size of the different convolutions; and, then supposing that we had done this, that we have neglected to take into account the temperament of each individual, and the activity of the cerebral functions.

Our intention therefore at present is only to afford accurate observations of what we ourselves have examined and ascertained; and we now, in pursuance of this plan, shall proceed to give some other measurements of encephala, such as we have found them in idiots and lunatics.

1. Gobinot, 24 years of age, and of middle stature. *Idiotism of the lowest grade:* no power of speech, but only inarticulate sounds, and cries. He seemed not to have even the natural instinct of hunger, and would not eat, unless induced to do it.

The encephalon weighed 1320 gram.
cerebrum . . . 1135
cerebellum . . 185

2. Inconnu, deaf and dumb, 43 years of age, and of a stature somewhat above the ordinary. *Idiotism of the lowest degree.* No speech, and scarcely any sign of intelligence.

The encephalon weighed 1370 gram.
cerebrum . . . 1205
cerebellum . . . 165

3. Darvoz, 39 years of age, and of middle stature. *Idiotism which places the individual below the brute.* No speech; sensations dull; a slight degree of memory; incapacity of clothing himself, or of defending himself against any external injury.

The encephalon weighed 1256 gram.
cerebrum . . . 1064
cerebellum . . . 192

4. Courtois, 46 years of age, and of middle stature. *Idiotism of the lowest degree.* Physiognomy almost simian. Incapable of answering to any question, or of taking any care of his person.

The encephalon weighed 1045 gram.
cerebrum . . . 907
cerebellum . . . 138
The anterior cerebral lobes . . . . 226

5. Boulot, 37 years of age, stature rather tall. *Idiotism of a very low degree.* The intellect is almost quite abolished; to all questions he answers, "ma tete, mon front sont egarés," or "dans la province de Joiny, ou a fait de moi un pauvre fou pour me perdre." He is often much excited and very turbulent. He has no regard to his animal feelings, and his life is almost entirely vegetative.

The encephalon weighed 1380 gram.
cerebrum . . . 1188
(its anterior lobes) 192
cerebellum . . . 300

6. Rollot, 46 years of age, and of large stature. *Idiotism very confirmed.* His intelligence was almost at zero. He said, for example, that he was only three years old, and it was only with extreme difficulty that he could pronounce a few words. His physiognomy was quite idiotic. He was employed at the Bicetre to turn the wheel of the great well there.

The encephalon weighed 1025 gram.
cerebrum . . . 896
(its anterior lobes) 250
cerebellum . . . 135

7. Cresson, 23 years of age, and of large stature. *Imbecility very strongly marked.* Speech embarrassed and stammering; physiognomy dull and heavy; although capable of manual work, never could be taught any art.

The encephalon weighed 1105 gram.
cerebrum . . . 920
cerebellum . . . 185

8. Mallebranche, 44 years of age; stature rather tall. *Idiotism quite confirmed.* Speech scarcely intelligible. Extreme chorea of all his limbs.

The encephalon weighed 975 gram.
cerebrum . . . . 825
(its anterior lobes) . 240
cerebellum . . . 150

9. Favelle, 57 years of age; middle stature. *Imbecility with irregularity of muscular movements.* He was admitted into the Bicetre as an idiot at the age of 12; and the only work, he was enabled to do, was to assist in turning round the wheel of the well. His speech and his walk were embarrassed and difficult. His arms were in a constant trembling. His intelligence was correct, but very inconsiderably developed.

The encephalon weighed 1235 gram.
cerebrum . . . 1077
(its anterior lobes) 300
cerebellum . . . 158

10. Chambin, 67 years of age, middle stature. *Imbecility only partial.* He was never, however, able to gain his livelihood by any occupation; and, in consequence of this, he was put into the Bicetre at the age of 24. His intelligence was very small, but correct, as far as it went. His speech was stammering and uncertain. Being capable of considerable bodily exertion, he was employed at the hospital to assist the servants in various ways.

The encephalon weighed 1365 gram.
cerebrum . . . 1215
cerebellum . . . 150

*Remarks.* If we compare the various measurements in the preceding ten cases, we shall find that the following may be given as the average weights of the encephalon and of its great divisions.

The encephalon weighed 1218 gram.
cerebrum . . . 1043
cerebellum. . . 165

It thus appears, as well as we may judge from a limited number of data, that the encephalon and its divisions are less weighty in idiotic than in healthy persons. If we examine the subject more attentively we shall find that this diminution of weight does not affect the cerebrum and the cerebellum quite alike; the latter part in the idiot being nearer the healthy standard than the former. The whole encephalon is about one-thirteenth less heavy; the cerebellum is about one-eleventh; and the cerebrum is only one-seventeenth.

Such are the general results deducible from the facts which we have detailed. But if we now pass on to the particular examination of the cases, we shall not find any correspondence between the weight of the encephalon or of its parts, and the amount of intelligence, or, in other words, the degree of mental imbecility of the person. Thus, in Gobinot, Boulot, and Inconnu the weight of the encephalon equalled or even exceeded that of the organ in the healthy individual, although these poor miserables were in the lowest degree of idiotism; and, on the other hand, in Rollot and Cresson, both men of large stature, the encephalon was less heavy than in the three former, although their intellects were decidedly less degraded.

Dr. Lelut closes his interesting communication by repeating the two general conclusions, which may, he thinks, be legitimately drawn from the premises; viz. that the encephalon is generally less weighty in the idiot than in the sane person; and secondly, that the deficiency of weight is in most cases more considerable in the cerebrum than in the cerebellum.—*Gazette Medicale.*

---

### REPORT OF THE ACADEMY OF MEDICINE ON THE TREATMENT OF TYPHOID FEVER BY PURGATIVES.

The report was drawn up by M. Andral in his own name and in those of MM. Louis, Bricheteau, Bouillaud, Double, and Bailly, on a work recently published by M. De Larroque, and submitted to the Academy.

A considerable period of time had elapsed since the appointment of the commission to examine into the merits of this work. The reason which M. Andral gives for this delay is quite satisfactory. He and his colleagues wisely thought that, on a question of practical medicine, the fair and legitimate mode of procedure would be to try experimentally the treatment which was proposed by the author, and to compare the results of their own practice with those which had been adduced by M. De Larroque.

Several of the members of the commission, therefore, engaged in this undertaking; and the following was the plan on which they sought to proceed. They wished to treat, at first, a hundred cases of typhoid fever by the purgative method; then another hundred by the use of antiphlogistics; and lastly a third hundred by " une methode à peu près expectante."

Having obtained these data, they would then be able to analyse and compare the different results, and thus hoped to arrive at an accurate and indisputable solution of the question for inquiry. " But," says M. Andral, " de telles recherches ne sauraient s'improviser."

It appears, therefore, from this admission that the decision of the commission was founded more on the preassumed opinions of the different members, than on any series of experiments instituted at the time for the purpose. With that judicious caution, for which M. Andral is justly celebrated, he prefaces his report by requesting that it may be viewed only as a " travail provisoire."

These remarks may be useful, as introductory to the following details.

" If the Pyrexia," says M. Andral, " which in the present day is called typhoid fever, is merely a gastro-intestinal inflammation, and if the numerous and varied symptoms which it exhibits during its progress are only the sympathetic effects of the primary irritation of the digestive passages, then the question as to its treatment must be of very easy solution: the antiphlogistic method, more or less actively pursued, is necessarily the only safe and scientific practice.

If, however, while we admit that typhoid fever has its " point de depart" in a phlegmasia of the digestive passages, we suppose that there is the peculiarity of the follicular apparatus of these passages being the special seat of the morbid action; and if, moreover, at a longer or shorter time after the first appearance of the lesion of the digestive tube, there manifest themselves a certain number of symptoms, which are not all considered (as the advocates of the former theory maintain) to be merely the sympathetic effects of the intestinal phlegmasia, but which are supposed to be owing to the absorption of the putrid matters which are deposited on the interior of the ulcerated bowels, and to the consequent poisoning and infection of the mass of blood, and therefore of the whole system, *then* the therapeutic problem is much more complicated and difficult of solution.

On these principles we can understand how that, independently of the antiphlogistic treatment, the physician will endeavour either to discharge the hurtful and absorbable matters contained in the bowels—and hence the utility of purgative medicines will be admitted—or to correct, if possible, the infection of the blood by means of tonics, the chlorurets, and so forth.

Lastly, if in typhoid fever the intestinal lesion be viewed only as one of the elements or symptoms of a disease, which, like genuine typhus* (vrai typhus), or like small-pox, attacks the whole organism of the body, we are forced to admit that the question as to treatment is placed upon a new footing.

The therapeutic principles must necessarily vary according to the views which are taken of the primary nature of the disease. If it be supposed that its nature or essential character is uniformly the same, and modified only in different cases by the accidental circumstances of age, constitution, weather, climate, &c., then most assuredly the same general principles of treatment must be applicable to all, or at least to most cases. Some writers maintain that a "sur-excitation" of the sanguiferous system is the predominant feature of typhoid fever; and by them the antiphlogistic or debilitant mode of treatment is recommended. Others, on the contrary, have supposed that an asthenic state, primary or consecutive, (to speak the language of the Brunonians) is the culminating pathological condition; and with them the chief indication in the treatment is to support the vital powers by means of stimulants and tonics.

Besides these two sects, the Sthenists and the Asthenists, there has been a third sect, whose followers have maintained that the most important pathological feature in typhoid fever is a vitiation of the fluids, in consequence of the presence in them of a morbific principle absorbed from the intestinal tube; and by these physicians, therefore, as we have already said, the purgative and antiseptic treatment has been especially patronised.

But whichever of these three doctrines may be adopted, it is apparent that the practice founded upon either of them must be to a certain degree uniform and exclusive. It may admit of change and modification, according to circumstances; but the physician, if sincerely convinced of the truth of his hypothesis, is in reason bound to base his practice upon its deductions.

Very different, however, from this mode of procedure are the views and precepts of the Eclectic school of medicine. According to the doctrines of this school, the judicious physician will not trouble himself about the proximate cause of the disease, which he is about to treat. He will be satisfied with studying and watching the symptoms in each individual case, and, unaffected by any hypothesis as to the primary cause of these, he will only direct his remedies to counteract or abate them. It is by following this path of investigation that Pinel and others have divided fevers into several families or classes, according to their prevailing type. Hence we read of the inflammatory, of the bilious, of the ataxic, and of the adynamic forms of typhoid fever, in the writings of this class of physicians. To each of these different forms an appropriate method of treatment has been proposed; and we are directed to pay especial attention to a variety of circumstances which influence and modify the type of individual cases; such as age, temperament, constitution of the atmosphere, the prevailing character of the epidemic, and numerous other physical and also moral influences. In many cases we are reminded that there are not any very distinctly-marked symptoms, these being rather negative than positive; and hence we are cautioned against adopting a practice "toujours agissante et perturbatrice," under such circumstances. It is to this set of uses that the "methode expectante" is peculiarly applicable; that method which is so beautifully alluded to in these words of Sydenham:

---

* It appears from this remark that many of the French physicians, and perhaps M. Andral is one of the number, regard typhoid fever as essentially distinct from "vrai typhus." We need scarcely say that this doctrine is not received by English pathologists. The typhus *mitior* and the typhus *gravior* are regarded as degrees of the same disease, which varies according to constitution, season of the year, character of the epidemic, and so forth.

"Natura enim sibi permissa negotium suum suo tempore exsequitur . . . ut nostrâ ope, nostris artificiis atque auxiliis non indigent; suis viribus optimè instructa, suis opibus locuples, suo denique ingenio satis edocta."

It is in one or other of these methods that physicians have treated the disease known by the name of typhoid fever. During the last 15 or 20 years, the antiphlogistic treatment has been almost universally adopted in France; and the chief difference in the practice of our physicians has consisted only in the activity in which this method has been pursued.

There have, however, been a few who have entered their protest against the universality of this practice, and who have assiduously maintained that numerous cases of typhoid pyrexia may be most advantageously treated by other means, and more especially by the use of purgative medicines. One of the most sagacious practitioners of this metropolis, whose loss the Academy has lately had to deplore, M. Lherminier, adopted this practice very frequently, many years ago, at La Charité.

The results, which he obtained, are detailed in M. Andral's own work on clinical medicine. Since his time, the use of emetics and purgatives has been tried by several physicians with various degrees of success. Of these M. Larroque has been perhaps the most assiduous. He has attempted to prove that, in almost all cases of typhoid fever, the daily use of some evacuant, either emetic or purgative, is very beneficial, whatever may be the lesions with which the fever may be complicated, the character of the symptoms present, or the temperament of the patient.

The work, in which he has recorded his observations, and which forms the groundwork of the present report to the Academy, is partly theoretical and partly practical. In the first, he endeavours to prove that a vitiated state of the biliary secretion acts an important part, if not in the production, at least in the keeping up of the febrile movement: "Vicieusement accumuleé dans les intestins, elle devient pour la membrane muqueuse digestive une cause trés-puissante d'irritation, et c'est ainsi que cette membrane peut se phlogoser, s'ulcerer," &c. He accounts for the lower end of the ileum and the cæcum being chiefly affected by supposing, that the depraved intestinal secretions have the tendency to lodge there; and he says that this lodgment is very generally indicated by a gurgling noise emitted on pressure over the part. The cerebral and other nervous symptoms, as well as the occasional alteration in the state of the circulating fluids, he attributes to the absorption of the vitiated intestinal fluids into the mass of blood.

These ideas, it will be observed, are nearly the same as those which were professed by Stoll in the last century. He too trusted chiefly to emetics and purgatives in his treatment of typhoid fever.

If the theory were correct, the practice would be perfectly judicious; but it cannot be denied, that it is almost quite gratuitous to assert that the bile in fever is so much vitiated as to become of itself the cause of the serious lesions in the intestinal tube, which are discovered on dissection. Besides this objection, it may be remarked that the intestinal folliculi have been found swollen and inflamed at a very early period of the disease, before the bile, it may be presumed, could have lodged sufficiently long to induce such a morbid change; and again, in several cases in which the use of active purgatives has been adopted from the very commencement of the symptoms, and in which therefore all the intestinal secretions must have been most effectually evacuated, the same train of phenomena—such as the disturbance of the function of innervation, and the alteration in the character of the fluids—has been observed during the progress of the disease, and the same lesions of structure have been found after death, as when the bowels had remained quite inactive.

But, setting aside all merely speculative objections, let us now examine the results of M. Larroque's experience in the treatment of typhoid fever by the use of purgatives. The number of cases which he has reported amounts to nearly one hundred. They occurred chiefly within the walls of the Hôpital Necker, of which M. Larroque is one of the physicians; and among them we find examples of all the grades of the disease, from the slighter form, which resembles "un simple embarras gastrique, ou une légère enterite," to the more serious form, in which a state of extreme adynamia is present. In all, without exception, the same method of treatment was pursued. Without regard to the type of the symptoms, the state of the tongue, &c., two grains of emetic tartar were administered at the commencement. On the following day a bottle of Seidlitz water was given; and this was repeated daily, as long as the pyrexia lasted. If this purgative disagreed with the patient, other laxatives, such as calomel, cream of tartar, castor oil, may be substituted. Whenever the febrile state has nearly subsided, M. Larroque counsels the use of mild tonics and light nutritious food. Throughout the whole course of the disease, the beverage is either lemonade or rice water.

Whatever complication may be coexistent with the fever, M. Larroque makes little or no modification of his treatment, except when he has reason to suspect the presence of pulmonary engorgement. In that case, he employs Kermes mineral in repeated doses along with the purgative remedies.

As to blood-letting, M. Larroque condemns it as altogether inappropriate to typhoid fever. There is scarcely an exception, he says, to the universality of this remark. M. Andral, however, expresses his surprise at these statements in the following terms:

"M. Larroque cannot be ignorant that many cases of typhoid fever have been combatted by sanguineous depletions with a boldness which has been justified by incontestible success. Hence it follows, that if the utility of blood-lettings, rare or repeated, large or small, is not in the opinion of all sufficiently demonstrated; at least their general and absolute condemnation is not in accordance with the observation of facts."

To justify the evacuating treatment, as recommended by M. Larroque, he has published a table of 100 cases treated on this plan. Of these, ninety were cured; and in several of the fatal cases, the treatment was not commenced until after blood-letting had been previously used, or very unfavourable symptoms had set in.

Such success is, as M. Andral remarks, "fort remarquable." He is, however, too candid to question the accuracy or authenticity of the reported cases; and he alludes at the same time to the writings of some English physicians as in a great measure confirmatory of M. Larroque's statements; and also of M. Bretonneau to the same effect.

It deserves also to be mentioned that, since the first announcement of M. Larroque's plan, many of the hospital physicians in Paris have given a trial, partial indeed, to the purgative treatment. Of these, Dr. Piedagnel is the only one who has published his reports. He has treated 134 cases chiefly by the use of purgatives. In only a few cases he employed venæsection and emetics. Of the entire number he lost only 19. Although this success is not quite so great as that obtained by M. Larroque, it is still very highly satisfactory.

M. Louis, at La Pitié Hospital, treated 31 cases of *genuine* typhoid fever by purgatives. Of these 28 recovered. Of the three fatal cases, two belonged to the "cas graves," and one to the "cas moyens." Of the successful cases there were five "cas graves," seven "cas moyens," and fourteen "cas legers."

M. Andral himself also has given a very fair trial to M. Larroque's method. He treated 48 cases of what he considered indisputable typhoid fever. In 30 of the cases the symptoms were "très legers:" all did well. In eleven other cases the symptoms were much more severe, and indeed alarming; nine recovered, and two died. The remaining seven cases had already reached the ataxo-adynamic stage, before the purgative treatment was commenced. Six died.*

The mortality therefore in M. Andral's practice is greater than that in the cases reported by M. M. Louis and Piedagnel, and very much greater than in those of M. Larroque.

If we now take the whole number of cases, treated by these four gentlemen, they will be found to amount to 213. Of these, 40 proved fatal; thus giving the rate of mortality at rather less than one-seventh.

Let us now compare the preceding results of the purgative treatment with those of other methods.

Of eighteen cases, two-thirds of which were "grave," and which were treated by M. Andral partly with depletions of blood and partly with purgatives, six proved fatal—thus giving a very high rate of mortality.

Of twenty-seven other cases, two-thirds of which were mild, and which were treated with moderate blood-lettings exclusively, six only died. All these six cases were "graves," when the patients were subjected to the treatment. It would be unfair, adds M. Andral with great candour, to conceal that M. Bouillaud has met with much greater success with copious detractions of blood, than I have in my own practice.

Lastly of fourteen mild cases, which were treated with simple diluents and low diet, all recovered.

M. Andral, after detailing these particulars, cautions his readers very properly not to allow their minds to be too much biassed by these or similar arithmetical statements, as there may very possibly be little or no parity in the different sets of cases.

Returning to the consideration of M. Larroque's method, he repeats the declaration, that not a few very serious cases of typhoid fever are successfully treated by the daily use of purgatives, and that almost all the milder cases may be beneficially treated in this manner. The truth of this assertion is proved by most indisputable evidence, and cannot fail to suggest some useful considerations to the reflecting reader. All know that fifteen or twenty years ago many physicians in this metropolis (Paris) declaimed most vehemently against the use of all purgatives and emetics in any case of typhoid fever, contending that they were sure to aggravate the disease, and to convert the milder into the severe and fatal forms. These rash statements are now most clearly proved to be quite erroneous; and we have seen over and over again all the symptoms of the pyrexia gradually and very easily dissipated under the daily use of the Seidlitz water.

M. Andral acknowledges that these facts are not new to some physicians, (we suspect that he here alludes to the British school in particular) who have all along admitted the great utility of purgatives in typhoid fever; and, addressing his brother-academicians, adds: "mais à la plupart d'entre nous elles apparaissent comme des faits inconnus et en quelque sorte etranges."

(Nothing can more convincingly shew the utter ignorance, on the part of most French physicians, of the past and present state of medical science in this country.—*Rev.*)

In conclusion M. Andral gives it as his opinion and as that of other commissioners, that M. Larroque deserves very high praise for having recalled the attention of his brethren to the above most important point in medical practice. He recommends his brother academicians, and medical men generally, to give a fair and dispassionate trial to the plan proposed, not only in numerous cases of one epidemic or during any single year, but in successive years, and in all variety of circumstances. He with great propriety alludes to the varying type or general character of different epidemics, and to the necessity which such a diversity ought necessarily to produce in treating the same disease

* We cannot but express our surprise at such *experimental* practice as this. Is it to be wondered at that patients, in an extreme degree of debility and exhaustion, should sink under the constantly-repeated action of evacuants of any sort?—*Rev.*

during different epidemics. Hence all exclusive and, as it were, specific modes of treatment must be at once unscientific and improper. "Nous reconnoissons," adds he, "avec la plupart des grands maîtres, qui nous ont précédés dans notre difficile et laborieuse carriere, que dans toute maladie, quel que soit son siege et quelle que soit sa nature, peuvent apparaitre certains etats generaux de l'organisme qui en changent la physionomie, en compliquant la nature et en modifiant la therapeutique." Thus, what with the modifying influences of age, sex, temperament, state of mental feelings, as well the weather, climate, and other less appreciable atmospheric influences, diseases which to the mere anatomist seem to be nearly or altogether alike, are very different in the eyes of the judicious physician.

(The admirable precept inculcated in the preceding two sentences cannot be too much insisted upon in the present day, when there is such an indiscriminate passion for attempting to reduce the phenomena of life, in health and in disease, to a few simple states of action. —*Rev.*)

In concluding his very able report, M. Andral takes the opportunity of cautioning his hearers against the errors of a too implicit reliance on mere numerical details, however exact they may be, in determining the value of any therapeutic means. He adverts to the circumstance, well known to the experienced practitioner, that a plan of treatment, which succeeds admirably well one year, or during one epidemic, may be insufficient or even hurtful during another; and he reminds us that there is no remedy, however absurd and ridiculous it may be, which cannot boast in the opinion, or at least in the statements, of some, great and manifold success in certain diseases. "The medical arithmeticians," says M. Andral, "have supposed that it was quite sufficient for all practical purposes if they merely collect and count an ample number of cases; whereas it must be quite obvious that they should count also the circumstances peculiar to each individual case. Now, as these circumstances are infinitely varied, it follows that, under a therapeutic point of view, we meet with very few examples of disease in which all their conditions are the same, and which therefore can be properly compared with each other." The concluding sentence of the report is so eloquent and beautiful in the original, that we cannot refuse ourselves the pleasure of submitting it to our readers in M. Andral's own words:

"Penetrés de ces principes, persuadés que l'esprit de doute est la sauvegarde de la science et la garantie de ses progrès, votre commission a l'honneur de vous proposer, tout en rendant justice au merite de M. Larroque, d'ajourner tout jugement definitif sur la valeur de son traitement, jusqu' à plus ample informé, et jusqu' à experimentations plus nombreuses et plus longtemps continuées; de le remercier cependant de ses interessantes communications, de l'engager à les continuer, parcequ' elles ne pourront etre toujours que bien accueillies, et de deposer honorablement son memoire dans les archives de l'Academie."

On the conclusion of the reading of the report, M. Bouillaud was the first who rose to address the Academy. He commenced his remarks by defining typhoid fever in these words: "An inflammation of the small intestine and of its follicles, with a *typhoid element*, the nature of which is not well understood, superadded to it."* He then proceeds to laud his favorite practice of blood-letting "coup sur coup" in almost all cases. Our readers, however, have of late read, in this Journal, so much of M. Bouillaud and of his practice in typhoid fever, that it would be an utter waste of time to go over the ground again. He acknowledges that he has not tried himself the purgative treatment, recommended by M. Lar-

* This definition is faulty in the extreme; the first member of the predicate being an evident assumption, the truth of which is far from being universally recognised, and the second being a mere statement of an unresolved difficulty. —*Rev.*

roque. His opinion, therefore, on this subject cannot have much weight. He says, however, that the very reports of M. Larroque afford a much less decided success, than those which he has published of his favorite practice of bloodletting. Of the entire number of cases alluded to by M. Andral, which were treated by the purgative plan, there was a mortality of one in seven or eight; whereas in his (M. Bouillaud's) cases the mortality did not exceed one in sixteen.

M. Piorry followed M. Bouillaud. He said that he quite agreed with M. Andral as to the errors which are apt to arise from trusting too much to statistics, or the numerical method, applied to medicine. No disease varies more in its peculiar complications and general character than typhoid fever; and in no disease, therefore, is it more necessary to vary or modify the treatment according to the circumstances of each case. In some cases the physician is obliged to draw blood, for the purpose of relieving congestion or inflammatory action; in other cases purgatives are imperiously called for, to evacuate offensive accumulations in the bowels, and to promote the flow of healthy secretions; in a third set the "methode expectante" will be found at once the most simple and the most efficacious. M. Piorry is, therefore, quite opposed to any single exclusive or uniform practice in the treatment of typhoid fever. "Le traitement general n'est autre que la collection des indications particulieres mises à execution." He illustrates his remarks by referring to the varying characters of pneumonia according to the cause which has produced it, and the other morbid states of the system with which it may be complicated.

There are no fewer, says he, than eight different species of the disease. 1. The traumatic. 2. The simple and regular, connected with a plasticity of the blood. 3. That which follows bronchitis, bronchorrea. 4. That which results from the action of poisonous substances. 5. Or, from the absorption of purulent matter into the system. 6. Or, from the stagnation of the blood in the depending parts of the lungs, from long confinement to the horizontal position (the *pneumonie* hypo-*statique* of our author). And, 8. The pneumonia, which is occasioned by a mechanical obstruction to the circulation. Now these eight different kinds of pneumonia are apt to be associated together in statistical tables, as if they were all of the same character, and all required exactly the same method of treatment. So also it is apt to be with the statistical tables of typhoid fever. M. Piorry closed his speech with the following very sensible remarks. "I always pay especial attention, in each individual case, to the phenomena of causality and symptomatology, which the patient may present; I endeavour to ascertain the organo-pathological states which may exist, and strive to combat them as they arise. The *ensemble* of the separate indications constitutes in my opinion the most prudent general treatment; and this again must vary according to season, locality, climate, prevailing character of each epidemic, &c. I am decidedly opposed to the practice of employing "une methode exclusive et banale, quels que soient les resultats d'additions, dont les unités ne seront pas bien precisées."

M. Castel then addressed the Academy. He also professed himself much opposed to the "ennui des chiffres, et la tyrannie des statistiques." He had a higher opinion of the "methode expectante" in the treatment of typhoid fever, than of any other general plan; although he was quite willing at the same time to admit the utility of purgatives, bloodletting, &c. in individual cases. He very justly contended that the fever has a certain course to pass through, certain phases to experience, and a certain "jugement" to undergo.

Now too-frequently repeated, or too active evacuations must necessarily thwart and obstruct Nature in her operations, and stifle, as it were, that very reaction, without which a safe cure cannot be effected.

The *bleeding* system and the *purging* system are both equally liable to the same objection. They are far too exclusive; and their advocates do not pay sufficient attention to the peculiarities of each case—peculiarities which manifestly require modifications of treatment. M. Castel says that he is friendly to the use of bloodletting, purgatives, and emetics, when there are obvious indications for their employment; but that he is decidedly hostile to the practice of following any particular method in all cases. In conclusion, he particularly condemns detraction of blood, whenever the fever is accompanied with diarrhœa—a complication, it is well known, of very frequent occurrence.

Here the discussion closed for the evening. It was resumed at the next seance of the Academy. The president for the evening, M. Renauldin, announced that he had received a letter from M. Larroque, in which he particularly desired that the members would not limit their discussion merely to the method of treatment recommended, but would examine the etiological question, whether the entero-mesenteric inflammation is the cause of the typhoid fever, or whether it is the result of the irritating unhealthy secretions which are poured into the intestines—the doctrine which he advocated, and on which is based the purgative treatment.

M. Rochoux was the first speaker on the second evening of the discussion. He professed himself to belong to the Eclectic class of physicians—that class which refuses to acknowledge any one particular or exclusive doctrine, as to the etiology and treatment of typhoid fever. He called in question the correctness of that favorite idea of so many of his colleagues, that the lesion of the intestines was the cause, or "point de depart" of the disease; and he therefore did not hesitate to condemn the uniform adoption of antiphlogistic remedies. He urged upon his hearers the importance of attending to the following circumstances in the treatment of typhoid fever: the dynamic state of the vital powers, the nervous condition of the patient's system, the state of the fluids, the state of the liver and other digestive organs, and lastly, the reigning or prevalent constitution. The ever-varying intensity of these different agencies must necessarily influence the character and severity of the symptoms, and must necessarily so modify the disease that, without destroying its unity, it will require great difference in its treatment. Typhoid fever is always a very complex disease, which becomes still more so in certain epidemics, and which demands the greatest care and discrimination for its management.

With respect to the practice of repeated bleedings recommended so earnestly by M. Bouillaud, M. Rochoux states that, although he is quite unwilling to question the accuracy of his statements with respect to his great success, he himself gave a fair trial to the practice many years ago, when the opinions of M. Broussais were dominant, and was obliged to abandon it in consequence of its injurious effects. It increased the nervous symptoms, the subsultus tendinum, the delirium, &c.

The same remark is applicable to other diseases as well as to typhoid fever. Thus M. Rochoux has met with cases of pneumonia in which, after the second or third bleeding, the patient's strength has suddenly become exhausted, and he has died as from asphyxia. The same occurrence he has noticed in several examples of erysipelas with delirium.

M. Bousquet then "prenait la parole." He admitted that all fevers, be their characters what they may during life, exhibit on the dissection of the patient nearly the same lesions of the intestinal canal. But while he made this admission, he was most willing to allow that there is great diversity of symptoms, and that there ought to be great diversity of treatment. He acknowledged that "la semeiotique est frequemment sacrifiée à l'anatomie pathologique." He is therefore quite opposed to the adoption of any exclusive or universal method of treatment.—"Un homme," says he, "arrive dans un hôpital avec une fievre continue. Si son etoile le dirige sur Necker (the hospital where M. Larroque practices), il sera purgé, purgé, purgé; sur l'hôpital de la Charité (M. Bouillaud) il sera saigné abondamment et coup sur coup. Faute des deux cotés. D'une part, toutes les fievres continues ne demandent pas les evacuans des premieres voies; et de l'autre, toutes n'exigent pas la saignée." M. Bousquet, like most of his predecessors in the debate, questions very much the utility of those registers, or statistics of cases, which are adduced by M. Bouillaud and others of that school, to prove the superior advantages of any particular line of treatment, especially during any single epidemic. Lastly, he expresses his opinion that the practice of the judicious practitioner will vary according to the symptoms of each individual case, and that he will not bind himself with the fetters of any special hypothesis.

M. Louis then addressed the Academy. He defended, against the attacks of most of his colleagues who had preceded him, "la statistique appliquée à la medecine." He considered it, he said, as "l'espoir de la science," and adduced some arguments, drawn from the history of other diseases besides typhoid fever, in favour of the plan. It is however unnecessary to enter upon this theme at present. The readers of this Review are probably well aware, that we have not so high a value for the numerical collection of cases as M. Louis and M. Bouillaud have. The numerical method applied to pathological anatomy is excellent; applied to therapeutic medicine, it is apt to be fallacious and inaccurate. M. Louis, it appears, did not enter upon the immediate subject of the discussion—the treatment of typhoid fever. We shall therefore pass on to the next speaker.

M. Bricheteau, one of the colleagues of M. Larroque, at the Hôpital Necker. "Although," said he, "typhoid fever has always its seat in the follicles of the intestinal canal, we must confess that it exhibits great diversity in its symptoms. M. Bricheteau is on the whole not friendly to the numerical method. He mentioned that when the "entero-mesenterique" fever appeared upwards of twenty years ago in the wards of the Hôtel Dieu, the *internes*, of whom he was one, used to meet together to canvass among themselves the merits of the different remedies, which were employed by the physicians of that great hospital. And what was the result of these discussions? "la mortalité etait à peu près la meme de toutes parts!" The late indefatigable M. Dance followed out this examination with great zeal, and the result of his enquiries was, that he found that the antiphlogistic and the tonic plans of treatment were both equally defective. He therefore gave the preference to the "methode expectante."

M. Bricheteau however confessed himself to be very decidedly favorable to the practice recommended by M. Larroque. He has imitated it in his own wards, and has experienced similar success; and he adduced the testimony of M. Bonneau, one of the physicians of the Hôpital des Enfans Malades, in favour of the use of purgatives in the typhoid fevers which he had treated there, during the last three years. —*Bulletin de l'Academie.*

*Remarks.*—We have no intention of prolonging this article by any observations of our own. But we cannot but express our surprize at either the ignorance or the unfairness of the French Academicians, in not once alluding to the labours of Dr. Hamilton of Edinburgh, and of other British physicians, in this most important department of medical practice.—*Rev.*

---

### DISCUSSION AT THE FRENCH ACADEMY ON THE NATURE AND TREATMENT OF GLANDERS IN MAN.

M. Rayer, the well-known author of the admirable work on Cutaneous Diseases, and one of the physicians of La Charité Hospital, communicated the following case.

A groom was in the habit of sleeping in a stable, where a glandered horse died. When he was admitted into the hospital, he presented a pustular eruption on the skin, and in the nasal fossæ and larynx; there were ecchymosed and gangrenous patches behind the ears, on the glans penis, and on the feet; indications of small abscesses in the lungs, and of large purulent collections in the substance of the muscles; and lastly, there were other symptoms usually grouped together under the name of typhoid. The man died; and on dissection the following morbid appearances were discovered. The cutaneous pustules, when examined, were not unlike to those seen in cases of ecthyma or of variola. Similar pustules were found in the nasal passages and also in the larynx. Numerous vomicæ were detected in the substance of the lungs, and in several muscles there were large abscesses filled with a sanguineous purulent matter.

These are the very lesions which are discovered in the bodies of horses, that have died of genuine glanders. We cannot therefore refuse to admit, that the case now detailed was one of the same disease, occurring in the human subject. But in order to prove indisputably the correctness of this assertion, M. Rayer inoculated a horse with the matter of the pustules from the groom, and the result was that the horse became affected with glanders.

M. Dupuy, one of the most distinguished veterinary surgeons in Paris, and attached to the great horse-establishment at Alfort, addressed the Academy to the following effect. He recognised two species or forms of glanders (morve), the chronic and the acute. The former is truly a tubercular disease, affecting chiefly the substance of the lungs. The tubercles are in a greater measure composed of a chalky matter, as has been proved by the beautiful experiments of M.M. Thenard and Lassaigne. There is therefore a tubercular diathesis in the bodies of the lower animals, as well as in the human subject. M. Labillardiere has analysed the milk of a cow, which was affected with tubercles in the chest, and he discovered that it contained a much larger proportion of phosphate of lime, than in health. When this cow died, an attempt was made to preserve the skeleton; but this was found impracticable, in consequence of the extreme friability of the bones.

Sometimes the tubercles commence as hydatids. A chalky secretion takes place from the interior of the cysts, and ultimately they are converted into solid tubercles.

The chronic form of glanders, such as we have now described, is not contagious. The acute form, on the other hand, is highly so. It has no resemblance to, or analogy with, the chronic species; and indeed it ought not to be called by the same name. It is much more similar to the rot or scab of sheep; and this latter may in many respects be compared to the small-pox in man. Such at least is my (M. Dupuy's) opinion, although I know that many veterinary surgeons disagree with me. We have met with several cases of a disease in the horse, which is very closely allied with the rot in sheep, as well by its exterior and interior characters, as by its symptoms and the morbid lesions found after death. We have given it the name of "*eruption claveleiforme.*" (The word "clavelée" is the French for the "rot.")

With respect to the acute form of glanders, I deem it a disease essentially eruptive, and highly contagious. He proposes to designate it by the appellation of "*coryza gangrenosa.*"

M. Barthelemy rose next to offer his sentiments on this curious subject. He alluded to the category of symptoms, in such a case as that related by M. Rayer, as indicating a disease quite distinct from any in the ordinary nosological catalogues. The three leading symptoms are the nasal flux, the tumefaction of the submaxillary and cervical glands, and the appearance of ulcers on the pituitary membrane. It usually runs its course in from five to twelve days. From the commencement of the disease, there is a general malaise, feverishness and disturbance of all the vital functions; the nasal discharge consists of a serosity mixed with a viscid matter, which is at first of a yellowish and subsequently of a red colour, bloody and very fetid; the pituitary membrane is red and afterwards purple or even quite black; it swells, becomes much thickened, and then ulcerates; the breathing through the nostrils is difficult and snorting, and sometimes the dyspnœa in consequence is so great, that the animal dies quite asphyxiated. The cervical ganglia are much enlarged and very painful on pressure. When the glanders is complicated with the farcy, tumors of the size of a walnut or thereabouts make their appearance in the subcutaneous cellular tissue. Some of these are single or isolated; while others form clusters around the large lymphatic vessels, which follow the course of the principal subcutaneous veins. These tumors, of a soft consistence from their first appearance, break in course of time, and form ulcers; the matter which they contain is viscid and red; the animal becomes rapidly weaker and more emaciated, and speedily dies. On dissection, the pituitary membrane is found in the state we have described above; it is softened, and partially destroyed either by large ulcerations or by gangrenous eschars.

The chronic form of glanders is very different. Its course is so slow that, were it not for the presence of the three characteristic or pathognomonic symptoms, the affected animal might seem, for months or even for two or three years to be in moderately good health. It preserves its appetite, embonpoint, strength and liveliness, and it may be as fit for work as any other horse. The nasal discharge, usually from one nostril, is the first symptom which attracts notice: sometimes, however, this is preceded by a tumefaction of the sub-lingual lymphatic glands. The discharge is at first scanty, and of a whitish colour; it adheres to the nostrils, and partially plugs them up. Perhaps the disease does not make any visible progress for a length of time. It may appear to be stopped for some months. Again the symptoms return; and then probably the nasal membrane is found to be partially ulcerated.

The chronic glanders is sometimes complicated with farcy; the acute form is very generally so. The farcy is, properly speaking, a disease essentially of the lymphatic system. It is characterised by the presence of the subcutaneous tumors, which we have described above. These tumors are usually hard and indolent at first, and afterwards become soft and painful. Some are transformed into ulcers: the matter is usually white, of a thick consistence, and inodorous. Those veterinary surgeons, who regard chronic glanders as non-contagious, view the farcy in the same point of view. Farcy is certainly a much less fatal disease than glanders. Out of a hundred horses affected with the former, the surgeon may expect to cure between seventy and eighty; whereas it is very rare that a cure of genuine glanders ever takes place.

M. Barthelemy then proceeds to consider the question of the transmissibility of glanders from the horse to the human subject. He is not inclined to believe in this opinion. The glanders he considers as a disease essentially and exclusively affecting the Solipedous animals, such as the horse, ass, mule, &c. He says that if a glandered horse is kept with cows or sheep in a stable, the latter have never been known to be affected; and he also alludes to the extreme infrequency with which men, who are constantly engaged in the stables of glandered horses, are ever affected with analogous symptoms.

At the vast veterinary establishment of Alfort, where upwards of 600 glandered cavalry horses were sometimes collected during the war, none of the pupils or other attendants were ever known to be affected.

From all this, he is very doubtful as to the propriety of regarding the disease in M. Rayer's patient as genuine glanders. The man had led an irregular debauched life, and had ruined his constitution by all sorts of vicious indulgence. M. Rayer states in his report that the nostrils were dry; there could not therefore be any nasal discharge. Neither does it appear that the cervical lymphatic glands were swollen and inflamed. As to the gangrenous vesicles observed in the body, we certainly meet with these occasionally in the horse; but they are only of occasional or accidental occurrence (epiphenomenes), and not a characteristic symptom of the disease. The morbid appearances found on dissection also were not very distinctly those of genuine glanders. The pituitary membrane was not thickened, softened, black, nor ulcerated, as it is usually in a glandered horse.

In fine, M. Barthelemy expressed himself to be very doubtful as to the true character of the disease in M. Rayer's patient; and he confessed that he was not yet convinced that the genuine glanders of the horse is ever transmitted to the human subject.

M. Rayer replied to the objections of M. Barthelemy. He said that the horse, which died in the stable where his patient had slept, was affected with the genuine acute form of glanders, and that it had been sent to the *esquarrissage* (Anglice, the knacker's) on the tenth or twelfth day of the disease.

He did not quite agree with M. Barthelemy in regarding, as pathognomonic of genuine glanders, the three symptoms of nasal discharge, tumefaction of the submaxillary glands, and ulceration of the pituitary membrane. All these symptoms are present, in a greater or less degree, in the chronic form, which most veterinary surgeons, and M. Barthelemy among the number, carefully distinguish from acute or genuine glanders. M. Rayer distinguishes three forms of the latter: one is accompanied with ecchymosis and gangrene of the pituitary membrane, with sanguineous infiltration of the larynx, and with hemorrhage, and patches of inflammation in the substance of the lungs; another with an eruption of pustules and with ulceration of this membrane; and the third with both these morbid states united.

"And do not," added he, "suppose that I have improvised these distinctions: they are drawn from nature, and you will find their elements in the Treatise on Acute Glanders of our distinguished colleague M. Dupuy."

He proceeded to state that he had himself seen, more than once, in horses which had died from genuine glanders, ecchymoses, gangrenous patches, pustules and ulcerations in the nasal passages, pustules on the skin, collections of pus in the subcutaneous cellular tissue, ecchymosis, and, lastly, hepatized and purulent points in the substance of the lungs. Now these are the very lesions which were discovered in this patient, whose case he had read to the Academy. He again alluded to the very convincing argument drawn from the result of the inoculation of a healthy horse with the matter taken from his patient. This horse died (or was killed) on the twenty-first day after the inoculation was performed, and the following lesions were found on dissection, in the presence of several distinguished friends. There were several pustules in the nasal fossæ; large ulcerations at the edges of the nostrils, and smaller ones on the septum narium; farcy ulcerations near the eyelids, consecutive to pustules developed in the subcutaneous tissue; minute points of hepatization and of ecchymosis in the substance of the lungs; and lastly an inflamed and suppurating condition of the submaxillary glands and lymphatic vessels.

With such facts as these before us, we are bound to believe that our patient died of genuine glanders.

M. Bouley then addressed the Academy. He commenced his remarks by drawing a distinction between the chronic and acute forms of glanders and of farcy. Chronic glanders, he said, is a tubercular affection, which has its chief seat in the pituitary membrane. It is peculiar to the solipeda. It commences obscurely, and its progress is very slow at first. The earliest symptoms are either slight tumefaction of the sublingual lymphatic glands, or a whitish-coloured discharge from one of the nostrils. These symptoms gradually increase in severity. The glands become more swollen and painful; the discharge is more copious, assumes a yellowish colour, and adheres to the edges of the nostrils; the pituitary membrane becomes discoloured, and begins to exhibit numerous white specks, which on examination are found to be tubercles in a state of crudity. At length, after a lapse more or less considerable of time, the tubercles soften, and ulcerations develop themselves on the mucous membrane of the nose.

As long as the glanders is local, that is to say, confined to the nasal cavities, the horse may retain every appearance of health, and may be as fit for work as ever, in point of strength at least.

On dissection, the nasal mucous membrane is found of a yellowish colour, thickened and covered with a greater or less number of deep ulcerations, which are usually most numerous along the venous sinuses, the alæ nasi, and the lower part of the spongy bones. The veins are ordinarily filled with a purulent matter, and here and there we observe crude or softened tubercles. Lastly, the lymphatic glands are found to contain a softened tuberculous matter —a phenomenon which we never discover in the acute form of glanders, and which alone suffices to distinguish it from the chronic.

The chronic form of farcy is not quite so well known as that of glanders. According to some, it consists in a chronic inflammation of the lymphatic vessels; according to others, in a peculiar morbid alteration of the lymph itself. It manifests itself by the eruption of tumors, more or less circumscribed, and of various forms. These are always situated along the *trajet* of the lymphatics, and are produced by the inflammation of the cellular tissue surrounding them. The tumors, which are at first painful, become in the course of the disease cold and hard: they continue for some time in this state, and at length become soft. The purulent matter, which they are found to contain, is of a whitish hue, and of a peculiar oily aspect, characteristic of the disease.

The wounds resulting from the opening of these tumors shew do disposition to heal, but quickly assume all the characters of genuine ulcers.

In conclusion, chronic glanders and farcy so closely resemble each other in many features, that one is often the prelude of the other. Farcy, however, is usually less serious than glanders; the latter being almost always incurable.

Let us now briefly consider the two diseases which have been improperly designated acute glanders and acute farcy.

Under the former designation, veterinary surgeons have been long in the habit of confounding two affections, the acute glanders properly so called, and the gangrenous glanders. The first of these forms consists in a subacute inflammation of the pituitary membrane, with a development of pustules, which ulcerate and give rise to a more or less copious discharge. It is always accompanied with a painful engorgement of the lymphatic glands of the throat, is highly contagious, and almost invariably terminates fatally.

In the early stage of the disease, besides the "tristesse et l'abattement" of the horse, the pituitary membrane is observed to have a yellow colour. There are next seen on this membrane pustules, which are red at the base and white at their tops. A yellowish and sanious discharge flows from both nostrils, and the lymphatic glands in the neighbourhood become swollen and painful. After the lapse of a few days, the pustules ulcerate; the discharge becomes more abundant and more glutinous; the nasal passages become more swollen, and the respiration in consequence is difficult and labouring. While these morbid changes are going on, the skin also in many cases becomes the seat of numerous papulæ or small tumors, which speedily soften and suppurate. The strength of the animal is rapidly exhausted, so that death usually occurs in from eight to twelve days.

In such cases we find on dissection deep ulcerations, which are surrounded with red areolæ; the pituitary membrane is in a great measure destroyed; the sinuses and hollows of the spongy bones contain a bloody purulent matter. We find also sometimes similar pustules on the mucous lining of the larynx and of the trachea. The lymphatic glands are red and infiltrated with a serosity; and the subcutaneous cellular tissue also is infiltrated partly with serum and partly with effused blood.

There cannot be a doubt that this form of glanders is highly contagious. Chronic glanders is certainly not. But if on this latter form there should supervene the acute form, then decidedly the case will be found contagious. It is probably by not having attended to this complication, that some veterinary surgeons have asserted that chronic glanders is contagious. According to our experience it is not, unless complicated with the acute form.

The second or gangrenous form of acute glanders,—the *coryza gangrenosa* of M. Dupuy—has been very generally confounded with the form just described. The animal is, from the beginning of the disease, dull, weak, and tottering; the pituitary membrane and the conjunctivæ of the eyes are "pointillées en rouge;" and the limbs, scrotum, nose, &c. present an œdematous engorgement. After the lapse of 24 or 28 hours, the spots on the nasal membrane widen and extend, and they at the same time assume a livid colour. The engorgement of the limbs increases, and the animal begins to discharge a yellowish matter mixed with streaks of blood. This state of things continues for two or three days. Then the pituitary membrane becomes more livid, and soft, and throws off gangrenous eschars, under which are found deep ulcerations. The discharge is still more copious, and exhales a most fetid gangrenous smell; the respiration becomes more and more impeded in consequence of the increased tumefaction of the nasal passages, and death speedily ensues. On dissection numerous patches, gangrenous, ulcerated, and ecchymosed, are found along the pituitary membrane; the subjacent tissues are more or less deeply disorganized, and numerous petechiæ, or soft gangrenous patches are discovered in the trachea and also in the substance of the lungs.

This form of the disease, in which the circulating fluids are very obviously much altered, is contagious like all carbuncular affections.

So much for the two forms of acute glanders. Let us now very briefly consider the characters of acute farcy. This, like its chronic form, has its seat in the lymphatic vessels; but it proceeds in its course much more rapidly, and moreover exhibits very different symptoms. It commences with a slight eruption, under the form of cords or streaks, which disappear and then return. The papulæ or small tumors gradually increase in number over different parts of the body, and remain persistent. At the end of four or five days, they soften, ulcerate and penetrate into the subjacent tissues. Often at this stage of the disease, a similar eruption manifests itself in the substance of the pituitary membrane, and in some cases causes a speedy death by suffocation. This affection should not be confounded with the eruption, with which the acute form of glanders is often complicated. It seems to result from the researches of Delafond, the professor of pathology at the great veterinary school of Alfort, that the tumors which are developed in the latter disease, are strictly pustular, and have their seat in the cutaneous tissue, whereas those of acute farcy have their seat in the lymphatic vessels.

In conclusion, M. Bouley thus expressed his opinion respecting the disease, under which M. Rayer's patient laboured. He pointed out certain features, in which the symptoms differed from those of genuine glanders in the horse. His own words are: "nous sommes donc forcés de reconnaitre qu'il n'y a point parité entre la maladie de Prot, et la morve aigue du cheval; mais l'exactitude et la bonne foi veulent que nous convenions aussi que, si ces maladies ne sont point parfaitement semblables, elles ont du moins quelques points d'analogie qu'on ne peut nier."

He is very doubtful that the patient was infected by the horse, which had died in the stables; and he does not hesitate to say that he is not satisfied whether glanders be ever communicated from the beast to man. He points to the extreme frequency of the disease in large stables and in certain seasons, while the analogous affection in the human subject is of extremely rare occurrence amongst the grooms who are in constant attendance.

Finally he suggests to the consideration of the Academy, that it might be very well that the question as to the transmissibility of glanders from the horse to the human subject should be fairly and minutely discussed.

M. Velpeau was the next speaker.

He had seen M. Rayer's patient, and had subsequently examined several glandered horses. The result of his observations was that the man Prot had most certainly died of glanders.

As to the objections urged by M. Barthelemy and others against the transmissibility of the disease, M. Velpeau is not inclined to attach much weight to them.

The alleged circumstance of the disease being of very rare occurrence amongst the grooms of a stable, where there may chance to be a number of glandered horses, ought to be well sifted, before we are influenced by it. It is to be kept in mind, for example, that it is only within the last few years that the attention of medical men has been directed to this inquiry. While it was not even suspected that glanders could be transmitted from the horse to man, the profession heard little or nothing of the disease. Not knowing what to make of certain cases, it is more than probable that many practitioners have omitted to keep any reports of them, or if they did, the details were meagre and inaccurate. Need we say more on this subject, than merely remind our readers how long cowpox must have existed, before it was understood by, or even known to, medical men?

We must admit, indeed, that the disease of the man Prot did not exhibit all the phenomena of genuine glanders. There was not, for example, the peculiar, yellowish, and glutinous discharge from the nostrils. It is possible, however, that the secretion may have flowed backwards, and been rejected by the mouth; for he certainly "rendait par la bouche une matiere semblable à celle que rendent les chevaux par le nez;" and this supposition is not at all unlikely, when we take into account that the patient lay constantly on his back. The pustules which were found on the nasal and laryngeal mucous membrane were, it cannot be denied, very similar to those which we meet in a glandered horse.—*Bulletin de l'Academie.*

---

### On the Endermic and Inoculative Method of Treatment.

We shall first narrate three or four cases, and then append a few remarks.

Case 1. *Intermittent Fever treated by the Endermic Method.*

A man aged 38, was received into the Hôtel Dieu, on the 21st of March. He had an intermittent fever which had lasted seventeen days; the first attack was caused by witnessing the sudden death of one of his companions. The fever came on towards eleven and lasted till four.

The 25th March, at nine in the morning, two grains of sulphate of quinine were spread on a small blister applied to the epigastrium. The fit returned, but was delayed two hours and a half, and only lasted an hour and a half instead of five hours. Two grains more of sulphate of quinine were applied to the sore. The 27th and 28th March, no attack; sulphate of quinine was however given internally, and there was no further appearance of fever.

Another patient with a similar complaint was admitted into the Hospital the 21st April; two grains of sulphate of quinine were applied the 25th, three hours before the supposed time the attack came on. No fever appeared. Two more grains were applied the following day, and four grains the day after; the medicine was then administered internally, and the man was cured. In several other cases a stronger dose of quinine has been applied, and the effects obtained have been nearly similar. M. Chomel has always *concluded* by prescribing a few grains of sulphate of quinine to be taken internally.

Case 2. *Cancer of the Uterus treated with Acetate of Morphia applied Endermically.*

Madame Detty, aged 53 years, having all the attributes of perfect health; married at five and twenty; mother of five children; her labours were always severe; at the birth of the last child forceps were employed, and the infant was still-born. At the age of 51, she had a dartre (herpes) on the left fore arm; sulphureous baths were ordered, and brought on an irritation in the interior of the womb, and an abundant menorrhagia. The treatment at the hospital Necker proved fruitless. The womb was explored through the speculum, in the course of July, 1824; there was hard scirrhus unequal congestion on the neck of the uterus, it bled on the slightest pressure; there was a white discharge, with a fœtid odour; great difficulty in voiding the urine; the skin was yellow, and the flesh turgid and bloated. The patient grew worse in November, she was in agonies, rolled in her bed, and loudly invoked death as her only relief. Narcotics, the most powerful antispasmodics, strong doses of spirit of morphia, had no effect. The cruel sufferings were only allayed, and the blessing of sleep procured, by putting two grains of acetate of morphia on a seton. The application proved so soothing that her existence was prolonged till the 20th December, 1824, without any further sufferings. This observation proves that in cases where a cure cannot be hoped for, great advantages may nevertheless be found in external applications.

CASE 3. *Rheumatism cured by the Application of Acetate of Morphia to a Blistered Surface.*

A young man, named Choubert, aged 24, a baker by trade, was seized with such violent pains in all his limbs, that he could neither move nor sleep. The patient attributed his complaint to excess of fatigue in kneading the bread, intense perspiration, and sudden cold after his hard work. On the fourth day after the appearance of the disease, the pains settled in the shoulder, the arm, and left elbow, the limbs affected were perfectly motionless, a continual burning pain, the patient lost his appetite, and his countenance bore an expression of sorrow and suffering.

The 10th March, a blister was applied to the arm, but gave no relief. The 13th, the surface of the blister was covered with half a grain of acetate of morphia. Spent a good night, slight pain, no function disturbed. The 14th and 15th, same medication, same effect. The 16th, one grain of morphia, complete relief, no change in the functions, excepting slight contraction of the eyelid. The 17th and 18th, the applications having been omitted, the patient spent two bad nights, and suffered considerably. The 19th, one grain of morphia, the patient quite calm, but cannot raise his hand. The same means continued until the cure was complete.

CASE 4. *Asthma treated by the Endermic use of Musk.*

M. Loliot, aged 62, a farrier by trade, was admitted into the hospital Cochin, with an intense orthopnea; the sibilant rale was heard. The complaint was so violent that the patient remained seated, holding fast any object near him, his shoulders up to his ears, his head thrown back, mouth open, his neck stretched out, as if endeavouring to meet the air he longed to breathe. Bleeding having given only momentary relief, a draught containing six grains of musk was administered. Abundant perspiration, breathing easier, circulation less rapid; the dose was increased to ten grains, and the patient left the hospital cured. He went to his work for two months, at the end of which he returned to the hospital in the same state as before. A blister was applied to the arm, and at the expiration of a week it was covered with six grains of musk. A few hours after it was found to have precisely the same effect as if given internally,—abundant perspiration, after which increased relief, and rapid disappearance of all accidents. Cure followed.

CASE 5. *Nervous Headache cured by the Inoculation of Morphia.*

A woman, admitted into the hospital Beaujon, was very subject to nervous headache. On a former occasion she had experienced great relief by the application of a blister to the temple, the blistered surface being afterwards dressed with an ointment containing some muriate of morphia. Dr. M. Solon made trial of the endermic use of morphia in a different form this time. He made eight punctures in the temporal region with a lancet impregnated with a strong solution. The patient was speedily cured, but felt inclined to sleep during the day.

It is chiefly to the researches of MM. Lembert and Lesieur that the profession are indebted for having illustrated the good effects which may be derived from the application of various medicines to the skin deprived of its epidermis. One very great advantage of the practice is, that the most potent remedies may be sometimes used in cases, where the judicious physician might be unwilling to introduce them, even in the most minute quantities, into the stomach or rectum. Moreover the direct application of anodyne medicines to the seat of a painful affection will often succeed, when constitutional or general means fail of relief. It has been very well remarked—Why have medicines always something uncertain in their action? It is because they reach a surface ever varying in its condition, or the intestinal mucous membrane is irritated and covered with mucosities, or perhaps the stomach and bowels are full. In subcutaneous absorption, on the other hand, the absorbent vessels (and perhaps also the capillary veins) are always in a free state, when the skin has been recently deprived of its epidermis.

Hitherto the endermic, or, as some call it, the inoculative method of treating diseases, has not been much attended to in this country. The most potent and available remedies are perhaps the vegetable alkalies; more especially the salts of morphia, strychnine, veratrine, &c.

We are indebted for the facts in this and in two or three of the subsequent articles to the late numbers of the Continental and British Medical Review, edited by Dr. Rioffrey, of which we have already spoken favorably. We trust that Dr. R. meets with the encouragement, which he deserves.

---

### On the Action of certain Medicines on the Heart.

The following observations are from the pen of Dr. Lombard of Geneva.

1. *Assafœtida.* This is one of the most potent remedies against the irregularities of the heart's actions, when they depend upon a nervous or functional cause. Even when there is organic disease present, the internal use of assafœtida is often productive of very decided benefit, by checking, or at least moderating the palpitations, and by inducing a state of calm. When we prescribe the internal use of the medicine, we may administer it either in the form of pill or of mixture. Three grains or thereabouts, taken twice or thrice a day, will be a sufficient dose in most cases. The most convenient form of employing it externally is that of a plaster applied over the region of the heart. The formula recommended by Dr. Lombard is as follows:—

Assafœtida, ℥ij.

Gum ammoniac, ℈j.

Turpentine, gutt. vj.

Yellow wax, q. s.—Misce.

2. *Camphor.* Given in doses of from three to twelve grains has a very decided influence in moderating any violent action of the heart. It also assists the heart in expelling its contents, when it is overloaded with blood, and cannot easily discharge it at each contraction of its ventricles. Hence in many cases of dilatation, it is of great and very decided benefit. The following observations are so good, that we shall extract them in the author's words:—"This state of discomfort—from the heart not being able to expel the blood from its cavities, in consequence of their imperfect contractions—which is sometimes temporary, sometimes permanent, appears to be properly modified by camphor. A few days, even a few hours, have sufficed under this treatment to regulate the most violent ventricular contractions and shortness of breath, and irregular circulation ceases after the administration of a few grains of camphor. Is the action of this medicine sedative, or stimulating? This is a question on which I cannot presume to decide; but it is evident, after the researches I have made on the treatment of diseases of the heart, that care must be taken not to prescribe lowering medicines; and that the heart hypertrofied, but with obstacles at the orifice, or with dilatation of its cavities, must be considered as a muscle fatigued by the continual efforts requisite to maintain an equilibrium between the arrival and departure of this circulatory fluid; so that it *should be* strengthened, and its weakness counteracted by tonic medicines, and its action regulated by anti-spasmodic stimulants. Thence the indication of steel and quinine in the first case, camphor and assafœtida in the second."

3. *Digitalis*. The sedative action of this drug is, at best, most uncertain and unsatisfactory. Its successful administration seems to depend upon attention to a multitude of circumstances, such as the state of the stomach, the mode of living, the amount of the dose exhibited, and so forth. If the stomach is in a state of irritation, digitalis seems to exert little or no influence on the action of the heart. Under these circumstances it very often induces sickness, nausea, and even vomiting. If this disagreeable effect is induced, we should try to arrest it by effervescing draughts; but if these fail, the use of certain antispasmodics, such as æther, oxide of zinc, the nitrate of bismuth, or even opium itself, may be found necessary.

If digitalis is administered with the view of subduing palpitations of the heart, the doses should be rather large, than small and frequently repeated. A grain, for example, of the powdered leaves, or three spoonfuls of an infusion, (a scruple of the leaves to six ounces of water,) may be given three or four times a day. The infusion is certainly the most potent preparation, and the one on which we can best depend. It is however more apt to induce nausea than the dry powder in the form of pills. As respects the corrigents of digitalis, we find the following remark of Dr. Lombard. "What best succeeds to avoid or allay the symptoms of saturation is calcined magnesia, subnitrate of bismuth, subcarbonate of steel, and oxide of zinc. Several English practitioners have prescribed powder of calcined magnesia. I have always employed it with subnitrate of bismuth, so that I am unable to remark on its action when administered alone. Subcarbonate of steel is the best adjuvant of digitalis; to this medicine may be attributed the absence of accidents to persons who have taken digitalis daily, during several months. Oxide of zinc also arrests the symptoms of saturation of digitalis."

4. *Polygala Senega*. This drug is perhaps too little used by medical men. Taken as an infusion, says Dr. Lombard, it appears to diminish the circulation, and regulate the ventricular contractions.

In persons affected with diseases of the heart, dilatation of its cavities, polygala has corrected the irregularity of the heart's pulsations, and has lessened the sanguine stasis which seemed to threaten the dissolution of the patient. The doses given, vary from twelve to twenty-four grains of polygala in the course of the day; an infusion prepared with a drachm, and four ounces of water, has been given in four-and-twenty hours.

---

### Observations on the Cancrum Oris of Children.

To this very formidable disease of infancy various appellations have been given; such as *sloughing phagædena of the mouth*, *gangræna* oris, *cancer aquaticus* or *water-canker*, *gangrenous stomatitis*, &c. &c.

It occurs most frequently about the period of dentition. Sometimes however we meet with cases in children of from two to five or six years of age; and examples of a similar affection are occasionally met with even in adults.

The disease commences with a purple spongy appearance of the gum, either of the upper or of the lower jaw; to this state speedily follows ulceration, which eats deeper and deeper, until the alveolar processes are quite exposed. The ulcerated surface is much disposed to bleed, so that its character is often with difficulty ascertained.

In severe cases, the ulceration rapidly passes into a state of gangrene and sloughing; and these morbid changes extend from the gums to the lips and inside of the cheeks. The diseased surface becomes quite black, the teeth drop out, the jaw-bone becomes carious, and even the tongue may become partially destroyed.

In some cases, the disease commences in the lips or cheek, and extends thence to the gums.

The accompanying constitutional symptoms are usually an irritative feverishness, and a greater or less degree of diarrhœa, which quickly reduces the young patient's strength, and speedily carries him off.

Cancrum oris generally occurs in week, unhealthy, and ill fed infants. It is often a sequela of remittent fever, scarlatina, small-pox, &c. The disease is always dangerous, and very frequently fatal among the lower orders. In better life its progress may be often arrested.

As an example of the disease in its worst state we quote the following case from Dr. Hamilton's observations on the remittent fever and water canker, as reported in the Cyclopædia of Medicine. The child was five years of age, and had recently recovered from mild small-pox. The ulceration was reported to have existed only a week, although already it had made great havoc. It had extended from the gums to the palate, which, as well as the plates of the maxillary bone, was destroyed, so that there was a large perforation into the nose, the cavity of which was involved in the disease, the septum and spring bones being carious. The fetid acrimonious discharge from the mouth and nose produced ulceration of those portions of the skin with which it came in contact. In less than two days after, the cartilage of the nose was destroyed, and the disease had spread to the upper lip; it then continued progressively to involve hard and soft parts, until the nose, cheeks, eyes, and lips were successively destroyed. It had even extended to the brain, through the cribriform plate of the ethmoid bone and orbits, before the unhappy child fell a victim to the disease.

Some authors have erroneously compared cancrum oris to the scorbutic gangrene of the lips and gums, seen in genuine scurvy. But the analogy is not correct.

Young children very rarely are affected with genuine scurvy; and moreover, in cases of cancrum, we never observe the usually external symptoms of the former disease. In cancrum oris, the gangrenous ulceration is usually limited to one side of the face, or at least it affects one much more than the other side. In scurvy, on the other hand, the local disease affects both the upper and under jaw, and generally also both sides; the gums become tumid, and, as the disease proceeds, livid and disposed to bleed; a fungus shoots out covering the teeth, which do not fall out as in genuine cancrum oris, unless the sockets become decayed. Besides other local differences, the constitutional symptoms are very different in the two diseases. Cancrum oris is always accompanied with high irritation of the system, which is never, unless merely accidentally, present in genuine scurvy.

We have already stated that cancrum oris is infinitely more frequent in weak, ill-fed, and unhealthy children of the poorer classes, than in the higher walks of life. Dr. Hamilton was of opinion that certain localities, &c. predispose to the occurrence of the disease. It is more frequent in low, damp, fenny districts, than in higher and purer posisitions. It has been observed to be most malignant and fatal in warm weather after a rainy season, or when epidemic diseases of a putrid character, such as small-pox, measles, or angina maligna, have prevailed. It is especially fatal, when it occurs in children, who are recovering from any of these diseases.

From Dr. Hamilton's observations, it does not appear to be at all contagious.

With respect to the treatment, its success depends greatly on the progress which the disease has already made. When the ulcerative process is confined to the gums, a cure may be generally effected. Aperients and other constitutional remedies are almost always requisite, to subdue the constitutional feverishness and disturbance. When these are corrected, the local disease is often arrested, and the ulceration is found to heal spontaneously. Should it however become indolent, its surface may be touched with the oxymel æruginis, tinctura ferri muriatis, or with a mixture containing one drachm of muriatic acid and an ounce of the honey of roses.

When the disease assumes the character of sloughing phagedæna, our object must be to arrest the extension of the local mischief, and to support the powers of life at the same time. For the latter purpose, after we have evacuated the bowels gently with warm aromatic purges, we may give ammonia with vegetable bitters, syrup of sarsaparilla, and small doses of wine. Mild nutritious food, such as beef-tea, or any other animal broth, should be administered frequently.

The muriatic acid is the best local application. Combined with the honey of roses, it should be freely applied to all the diseased surface, and the mouth may be frequently washed out with a strong acidulated decoction of bark, to which some laudanum has been added. If the child cannot use a gargle, the mouth should be washed out with a syringe. The black wash is a good application when the ulceration begins to heal, or in the early stage of the disease.

In the worst cases of the disease, when it has extended to the bones, we cannot expect to be of much use. The pure muriatic, or nitric acid applied to the sore, and large doses of the carbonate of ammonia taken internally, are the best remedies we can use.

As illustrative of the disease, we are tempted to extract the following observations of M. Baudelocque, one of the physicians of the Hôpital des Enfans at Paris, as reported in Dr. Rioffrey's Journal.

Gangrenous stomatitis, or the gangrenous inflammation of the mouth in young children is very common in large towns, especially among the poorer classes. It is, therefore, by no means surprising there should be so many cases of it in the hospital for sick children. During the last six weeks, four affections of this kind have been treated by Dr. Baudelocque, and notwithstanding the danger of this disease, proved fatal only to one of the four children. We shall give a rapid sketch of the four cases, and shew the plan of treatment adopted by Dr. B.

On the 13th of September, a little girl, six years and a half old, was admitted into the hospital, with a cankerous affection of the mouth. This child had been ill for a week, and still had remains of the measle rash; the face was much swelled, the left side œdematized, the eyelids nearly closed: in the middle of the cheek there was a sore rather larger than a shilling; inside the mouth was an ulceration, and the whole depth of the cheek was invaded; lower down there was another sore; a dark discharge, of very fetid odour, flowed from the mouth; the gums were swelled and bleeding. Two large sinapisms had been applied to the legs during the measles, and there remained two large gangrenous sores invading the chief part of the teguments of the legs. Auscultation shewed that the lungs were seriously affected. The pulse was slow, scarcely perceptible, and a continual diarrhœa contributed to weaken the patient.

Although there could be but little hope of recovery, Dr. Baudelocque had the internal sore cauterized with hydrochloric or muriatic acid, and externally with a hot iron; the eschars were then to be sprinkled with the powder of chloride of lime. He also prescribed a mixture of a drachm of acetate of ammonia, and an ounce of syrup of quinine, two moderate injections of bark, and the sores of the legs to be powdered with the chloride of lime.

The cauterization was renewed several times. The following day the pulse was better, there was no swelling round the cauterized parts, and the face was less œdematized.

On the 26th, the cauterization was again made on the internal sore; four ounces of Malaga wine, to be taken by spoonfuls, were prescribed; the syrup of quinine, the acetate of ammonia, and other means indicated, were continued, but the young patient sunk, became delirious, and died on the 28th.

A post mortem examination was made the ensuing day. The periosteum of the whole side of the lower jaw to the articulation was detached. The lungs were hepatized in various degrees, and sprinkled with miliary tubercles; the digestive tube was empty.

There was not the slightest chance of saving this patient; the state of the lungs rendered all care useless.

The cauterization, however, seemed to have a good effect on the stomatitis, which made no farther progress, the fetid odour no longer existed, a degree of reaction had taken place, but the double pneumonia and excessive suppuration arising from the gangrenous sores on the legs, necessarily led to a fatal termination. In the other three cases this cauterization has been followed by a happier result, though one child was very young, and her state most serious.

A little girl, of five years old, had several gangrenous spots on the cheeks and upper and lower gums. This disease had also made its appearance during the measles. There was cough, fever, diarrhœa, and the running from the mouth was so fetid, that it was difficult to examine it.

All the gangrenous parts were touched with hydrochloric acid, and then powdered with chloride of lime. Syrup of quinine, tonic and antiseptic injections were prescribed. The fetid odour soon disappeared, and the gangrenous spots did not increase. The upper and lower teeth fell out with the sloughs, and a large splinter of the maxillary bone, so that after the child's recovery there was a double solution of continuity, which seemed to result from two semi-elliptic sections made in the upper and lower jaw.

This cure is undoubtedly very remarkable, and quite unexpected, for children of the same age taken into the hospital, and having any severe illness seldom recover.

The other two cases offer but little interest, after those just related. A little girl, admitted into the hospital, had been ill three months; there was a considerable swelling of the under maxillary ganglia on the left side. When the mouth was opened, several ulcerations of the cheek, the palate, and the tongue were visible; this affection appeared local. The ulcers were touched with hydrochloric acid, then covered with powder of chloride of lime. A gargle of honey and syrup of bark was prescribed.

The progress of this stomatitis was arrested after the second cauterization, and the young patient left the hospital cured, at the end of a week.

The fourth case is that of a little girl, who had two large ulcers in the cheek and on the tongue; the same treatment was followed as for the other child, and she seems to be in a fair way of recovery.

In the two latter patients, the stomatitis existed independently of any other disease, which does not often occur. This affection is almost always concomitant with an eruption, and especially with the measles, and there are generally serious lesions in the lungs, so that the stomatitis may be considered a dangerous complication, which hurries on the fatal termination.

The digestive tube does not in general shew very distinct marks of inflammation, and the purging is apparently owing to deglutition of the matter secreted by the gangrenous mucous membrane of the cheek. The preparation of bark, especially in union with opium, appears to be useful in moderating this diarrhœa.

---

GENERAL REMARKS ON THE DISEASES OF CHILDREN. By M. BAUDELOCQUE.

We have mentioned in the preceding article that M. Baudelocque is attached to the large Hôpital des Enfans in the French metropolis. The chronic and acute cases are arranged in distinct wards, and the younger children, under five years of age, are kept apart from the older ones. There is a particular ward for those who are affected with nervous or convulsive diseases, such as chorea, epilepsy, &c.

The class of chronic diseases which is perhaps more attended to than any other, is that of scrofulous, and also of cutaneous complaints. M. Baudelocque has devoted great time and labour to the study of these diseases, and the success which attends his practice is the best guarantee of his judgment and skill.

In reference to the general mode of treatment which M. Baudelocque follows in acute diseases of children, it deserves to be noticed that he is not friendly to the adoption of very active measures, except in a limited number of cases. Large bleedings, excessive purgation, or vomiting, he particularly deprecates; remarking, very justly, that in infancy and early youth there is, so to speak, such a strong tendency to health, that when it is disturbed even to a great degree, all that is generally required is to remove any impediments to Nature's own efforts, or at most to moderate their force, if excessive.

For the cure of bronchitis, angina, and in general all eruptive diseases, he trusts chiefly to quietude, low diet, and the withdrawal of all stimuli. Gentle purgatives and diaphoretics may be advantageously administered at the same time.

In hooping-cough, he seldom bleeds, unless there be very evident signs of pulmonic inflammation, or the patients be very plethoric and strong. He puts them on spare light food, exhibits small doses of oxide of antimony, and gives a cough emulsion. Under this simple treatment, most of his cases do well.

Even in scarlatina and measles occurring in young children, he seldom employs leeches. It deserves, however, to be mentioned that Dr. Baudelocque has recourse to depletory measures much more frequently and also to a greater extent in his private than in his hospital practice; the children of the poor being seldom so full-blooded and so active as those of the more wealthy classes. The following sentence is full of sound practical wisdom.

Dr. B. does not think an acute malady can be got rid of by profuse bleeding. Its intensity may be diminished, its complications decreased, but to arrest it suddenly by bleeding is not possible. In a typhus fever for instance, neither sanguine evacuations, nor purgatives, can arrest the progress of the disease; it must have its course, and if bleeding be not injurious, it is at all events useless.

With respect to the general plan of treatment pursued by Dr. Baudelocque in the chronic diseases of children, he has great confidence in the continued use of mild warm aperient medicines. He usually administers a purgative draught twice a week. If the child is taking any other medicine at the same time, such as iodine, arseniate of soda, &c., it is always omitted on those days on which the purgative is given.

The purgative plan of treatment—a dose being exhibited twice a week—may be persevered in for a great length of time, not only without any pernicious effect, but with the most positive and decided benefit. Certain diseases have been completely cured by this simple medication; in others the symptoms are very quickly amended, and then the cure is easily effected by other means. The favorite form of aperient medicine which Dr. Baudelocque administers to children is strong senna tea mixed with coffee, to which a little milk may be added. The young patients take it without reluctance, and it usually acts briskly on the bowels.

Manna or sulphate of soda are sometimes substituted for senna.

The oxymel of squills is one of the best and most convenient remedies for coughs and most pectoral affections.

The alterative medicines which Dr. B. most frequently uses are iodine, extract of hemlock, arseniate of soda, barytes, &c. The food allowed at the same time is ample and nourishing. Animal food, and even wine or malt liquor, are often quite necessary in cases of chronic disease, especially when the child is of a lymphatic and scrofulous constitution.

### ON THE TREATMENT OF GASTRALGIA, by DR. LOMBARD.

The medicine, which above all others Dr. L. has found useful in this painful affection of the stomach, is the subnitrate of bismuth. Even in cases where we cannot hope to effect a cure, as in organic disease of the organ, the sufferings are usually much soothed by the use of the subnitrate. Whether the disease be accompanied with vomiting or not, we may generally expect to obtain relief from it. However, like all other medicines, the subnitrate sometimes fails. Under such circumstances Dr. Lombard has occasionally succeeded with the oxyde of zinc. From six to twelve grains may be administered in twenty-four hours. Its action seems to be very similar to that of the subnitrate of bismuth; both appearing to exert a sort of sedative effect on the pneumo-gastric nerves.

The zinc seems to be especially suited to those cases of gastralgia, which are connected, or, rather we should say, accompanied with leucorrhœa. The uterine discharge is not unfrequently much moderated under the continued use of this metallic tonic. But whether this effect is produced or not, it cannot be disputed that that form of gastralgia, which we so often meet with in weak pale females, who have long suffered from fluor albus, is often very quickly relieved by the oxide of zinc, either alone, or combined with extract of hemlock, or henbane.

Those forms of gastralgia to which we have hitherto alluded are very generally connected with a certain degree of atony of the muscular powers of the stomach. Hence along with the pain there are usually other symptoms of imperfect digestion, such as anorexia, flatulence, and eructations, and so forth. We cannot, therefore, be surprised that the class of vegetable bitters and tonics is so often of marked utility in relieving the distress of gastralgia. These medicines may be servicably combined, either with the metallic preparations mentioned above, or with one of the alkalis.

The action of alkalis is very different from that of bismuth and zinc; and it often occurs that medicines belonging to this class succeed where metallic sedatives have failed. We have observed that pyrosis, acid vomiting, and gastralgia in all its forms, apparently dependent on acid on the stomach, generally give way when bismuth is taken. There are, however, some cases in which this medicament fails; then a chemical medication arrests the morbid symptoms; the use of soda, potass, magnesia and lime, often cures the different forms of gastralgia we have mentioned. The subcarbonate and bicarbonate of soda are most beneficial in cases of pyrosis and acid vomiting; they are also very useful in a species of gastralgia originating in the want of a sufficient quantity of salivary secretion.

Gastralgic symptoms are accompanied by dryness of the mouth in speaking, or after mental emotion, and particularly by the want of copious drink to assist mastication, and later on, digestion.

In these cases soda water, or the waters of Vichy, are most desirable; subcarbonate of magnesia, lime water, or a solution of caustic potass, answer the same purpose, in all cases where acidity is the predominant feature of gastralgic symptoms. The solution of caustic potass, administered in doses of a few drops in an aromatic infusion, has often succeeded when other medicines have failed; its action seems to be both chemical and tonic, and this combined property is precious in a number of cases where the acid secretion is caused by a debility, which tonics only can surmount.

There is a domestic remedy, which not unfrequently relieves gastralgia more speedily than perhaps any other; and that is a large draught of very hot water. A few drops of laudanum, taken in half an hour or so after the draught, are strongly recommended by the experience of some.

Every practical man is well aware of the influence of mental emotions on the state of the stomach generally; and hence we are prepared to expect that gastralgia should be not a little obedient to the agency of these. It is perhaps in consequence of this law, that antispasmodic medicines are often so serviceable in cases of gastralgia. Valerian and assafœtida are among the best. Hydrocyanic acid is sometimes a very useful medicine. We have not yet alluded to the use of purgatives in this disease. Whenever we have reason to believe that the intestinal contents have been retained, or are in any way vitiated, the exhibition of aperient medicines may be expected to produce good. In chlorosis, and other diseases accompanied with an atony of the intestinal functions, a prolonged course of aloetic purges, in union with small doses of mercurial alteratives, are perhaps the most efficient remedies. Gastralgia, being a not unfrequent symptom of these states, is relieved at the same time.

So much for internal medicines. External applications, however, are not to be neglected. We borrow the following remarks from Dr. Rioffrey's Journal.

Internal medicaments are not employed alone in gastralgia; external applications, either sinapisms, blisters, moxas, or soothing plasters, or morphia and prussic acid, are most beneficial in vomiting, and in cramps in the stomach. Cutaneous derivatives are a valuable resource when other methods of treatment fail, or act slowly; sinapisms are useful when there is great pain, but blisters are far more successful, particularly if suppuration be maintained by epigastric pomatum: blisters applied to the epigastrium have sometimes cured gastralgia, that existed for a considerable length of time. The most desirable effects have been induced by a blister on the nucha in gastralgia. which depends apparently on the morbid state of the brain or pneumo-gastric nerves; it seems more appropriate to apply the derivative as near as possible to the origin of the nerve; this physiological consideration has led me to place the blister on the nucha, and thus succeed in calming vomiting that had existed several months; gastralgic pains are also relieved by the method I now lay before practitioners as one of the most effectual for vomiting, either spasmodic or of any other nature.

The use of antispasmodics and narcotics, on the epigastrium, often succeeds in calming the most severe gastralgia. A plaster of assafœtida useful in cases of palpitation, is also efficacious in calming gastralgic pains, particularly when they originate in nervousness, and are relieved by antispasmodics. But whatever virtue assafœtida may possess, it is by no means equal to morphia and prussic acid, applied as frictions on the epigastrium; morphia and cyanuret of potass may be dissolved in water, in proportions of six to twelve grains of these salts, to three ounces of water; or they may be blended with lard—from three to six grains to one ounce—to form an ointment, which is then to be used in the endermic method.

---

### Case of Abdominal Tumor, which weighed 120 Pounds.

The patient, a man 58 years of age, had, with only occasional slight illnesses, enjoyed good health till about the end of the year 1833. At that time he began to be affected with hæmorrhoids. He was recommended by Dr. Guillon, his medical attendant, to leave these to nature, and not to attempt to stop the discharge of blood. He chose, however, to follow his own plan, and applied a strong astringent ointment, which had the effect of preventing in a great measure the occasional hæmorrhage. He became much fatter than he was before; but the chief increase in size was in the abdomen. Dr. G. accidentally met his old patient, six or seven months afterwards, and being struck with the disproportionate bulk of his belly, he requested him to permit a manual examination. There was no unevenness or irregularity to be distinguished, but an uniform tense swelling of the whole surface. On tapping the abdomen with the hands applied to the two sides of the abomen, a sense of fluctuation could be distinctly perceived. Dr. G. suggested the use of vapour baths, aloetic medicines, and the application of leeches to the anus, with the view of recalling, if possible, the hæ-

morrhoidal discharge. But his advice was not followed.

The size of the abdomen continued steadily to increase; but no inconvenience, save that from its bulk, was experienced. By the end of 1834, the abdomen measured more than four feet in circumference. In the right inguinal region there had appeared a tumor, of the size of an apple, which Dr. G. took at first for a bubonocele; but which he afterwards decided was caused by the enlargement of the lymphatic glands. On percussing the whole extent of the abdomen, the only part in which any resonance could be detected was in the left hypochondrium, where the stomach, distended with gas, was probably situated. At this time, the limbs and face of the patient were observed to be considerably emaciated, and every now and then there was an œdematous swelling of both ankles. Still there was no decided disturbance in any of the important functions of life.

After the lapse of another twelvemonth, Dr. G. saw his patient again. By this time he was so incommoded with the bulk of his abdomen, that he was obliged to confine his walks to the immediate neighbourhood of his house. He now tried the effects of drastic purges, digitalis, inunction of mercurial ointment, &c. But these means proving quite inefficacious, he submitted to the operation of paracentesis abdominis. The abdomen at this time measured three feet in length from the xyphoid cartilage to the pubes, four feet eight inches around at the umbilicus, and five feet and a half lower down. All the medical men, who were present, gave it as their opinion that the abdomen was filled with fluid. The trocar having been introduced to a moderate depth, it was withdrawn; but no fluid passed from the canula. The instrument was again introduced, and inserted deeper; but still no fluid flowed out. A probe being then passed along the canula, a resisting elastic object was felt. The idea of the case being one of encysted dropsy then occurred to the minds of Dr. G. and of his assistants. The abdomen was re-examined most attentively, and, as the sense of fluctuation was most distinct, it was determined to make a puncture on the other side of the linea alba. The trocar was introduced up to the hilt of its canula. But this operation was as unsuccessful as the other: no fluid escaped.

Three months after this unsuccessful attempt, the patient was seen by two other medical men of high repute, who, after a most careful examination of the abdomen, gave it as their opinion that there was a large quantity of fluid in the cavity of the abdomen, and that paracentesis should be again performed. It was proposed that the trocar should be introduced this time in the line of the linea alba, three or four inches below the umbilicus. But no better success followed this attempt. A long probe was then passed along the canula, to nearly a foot in depth, into the cavity of the abdomen. It communicated to the hand the sensation of numerous imperfect membranous partitions. It was supposed by some of the medical men that there was an immense accumulation of hydatids; while others confessed that they could not form any satisfactory conjecture as to the nature of the case. It was now proposed to establish several setons at different points of the abdomen, with the view of causing a copious suppuration. But this idea does not seem to have been carried into execution.

The condition of the poor patient became gradually more and more distressing. Not only was walking almost impossible; but now he could not lie on his back, for dread of suffocation. Often was he forced to rest on his knees and elbows for hours together, as he could not find ease in any other position. His general health too was much impaired; his limbs became œdematous; his strength gradually decayed, and death took place in the month of November, 1836.

On dissection the following appearances were discovered. The abdomen measured six feet five inches from the hypochondria to the pubes; three feet four inches from the xyphoid cartilage to the same point; and five feet two inches in circumference at the umbilicus. The sense of fluctuation was very perceptible. On the left side only was a resonance elicited by percussion. On laying open the abdomen by a long incision from the sternum downwards, the parietes were observed to be much attenuated and of a very pale colour. An enormous tumor was now discovered filling up the whole cavity. The colour of the mass and its partially lobed form made it resemble somewhat a monstrously enlarged liver. It adhered very intimately to the whole right side of the abdomen.

The tumor consisted of two masses, the upper one of which was of the largest dimensions. The lower one was denser and thicker; and in the hollow or groove between the two lay the mesenteric vessels. Both masses were attached in front of the vertebral column, extending from the pillars of the diaphragm down to the scrotum. The stomach, spleen, and the small intestines, had been pushed upwards and backwards.

The lower mass of the tumor occupied the greater part of the pelvic cavity, resting upon, but not adhering to, the vena cava, the iliac veins, and the sigmoid flexure of the colon. It measured four feet in circumference, its long diameter being nearly fifteen inches, and its shorter one more than seven inches. No trace of the omentum was to be discovered.

The consistence of the enormous tumor, of which we have attempted to give a description, was firm, and rather elastic and yielding. It communicated to the fingers the sensation as if hydatids were contained within. On cutting however into its substance, it was found to be homogeneous throughout, and of a uniform fibrous consistence. The pelvic portion had assumed in part all the firmness of cartilage.—*La Presse Medicale.*

---

### Ascites cured with the Inunction of Veratria Ointment.

The following case is taken from the report of St. Catharine Hospital at Stuttgart, and is published in one of the late numbers of the Medicinisches Correspondenz-blatt.

A young man, 17 years of age, was admitted into the hospital, with all the symptoms of a gastric fever, and with well marked ascites. The patient, whose aspect indicated a scrofulous constitution, had been recently exposed to frequent chills. The bowels were much confined, and the secretion of urine had for a length of time been very scanty.

A purgative, consisting of acetate of potass and extract of dogstooth, did not affect the symptoms. Small doses of extract of hemlock, of squills, and of cherry-laurel water, in conjunction with an infusion of junipers for the ordinary beverage, were administered for several weeks, but without any decided effect on the dropsical effusion. An ointment, consisting of four grains of veratrine and an ounce of lard, was then employed to an outward application. A portion, of the size of a nut, was ordered to be rubbed in upon the abdomen twice in the course of the day. After two days' use, the urinary secretion was found to be very considerably increased. The strength of the ointment was gradually increased, until it caused great irritation and convulsive movements of the muscles. Its diuretic effects continued very steadily; and at length the patient was pronounced to be quite cured.

It is to be observed that, notwithstanding the apparent success of the veratria ointment in the preceding case, it often fails entirely in dropsical cases. Two examples are related in the same report, from which we have extracted the previous details, where it was not of the slightest advantage.

---

### Abstract of the Report of M. Desportes on the Service of the Salpetriere and Bicetre Lunatic Hospitals.

The work of M. Desportes consists of two sections. The first exhibits a series of eighteen statistical tables, giving an account of the number of patients for each year, the name or character of each form of the disease, the occupation of the patient, the causes of the insanity, the relapses, the length of time each patient was under treatment, and lastly, the issue of each case. The second comprises the practical and philosophical deductions which may be drawn from these tables.

These tables are arranged in three classes. In the first class are grouped the cases, which were observed during the years 1825, 6, and 7; in the second, those observed in the years 1828 9, and 30, and in the last, those observed in the three following years, 1831-2-3.

*Number of Patients admitted, discharged, and cured, during Nine Years from* 1825 *to* 1833 *inclusive.*

| | |
|---|---|
| Admitted.......... | 8,272 lunatics. |
| Discharged cured ... | 2,763 |
| not cured | 1,863 |
| Dead ............ | 3,854. |

The relative mortality to the number of cases admitted may appear, at first sight, to be extremely high; but we are to remember, "que la mort frappe surtout les incurables."

The number of admissions during the last triennial period was greater, than in either of the two preceding periods. The cause of this increase is most probably to be found in the agitation of the public mind induced by the revolution of July, 1831. In the following year too, the epoch of the cholera, there were more admissions than usual. A similar increase in admissions was noticed also in private lunatic establishments, during these two years. In an article published by Dr. Belhomme in the bulletins of the Medico-practical Society, he has particularly alluded to the influence of stirring political and public events on the development of insanity. "It seems to me," says he, "well established, that not only great political events exert an influence on the development of insanity, but also that the number of insane persons must necessarily have increased in France during the last forty years, in consequence of the violent commotions, which have succeeded each other during that period. That such has been the case, is clearly proved by the uniform increase of admissions into our great lunatic establishments after any violent excitement of the public mind."

During the nine years, to which the report of M. Desportes alludes, the number of admissions of female patients has been more numerous by about one-fourth.

M. Desportes observes that, by a singular coincidence, the number of insane patients under treatment and in a state of convalescence, which amounted to 318 on the 31st December, 1827, and to 3,127 on the 31st December, 1830, was exactly 318 on the 31st December, 1833.

In what months of the year have the admissions been most numerous? The following is a table in M. Desportes' report. Of 8,272 admissions during nine years, there were—

| | |
|---|---|
| 281 | during June. |
| 268 | .. July. |
| 255 | .. May. |
| 237 | .. April. |

From this table it would appear that the month of June, when the hot season commences, is the period of the aggravation of insanity.

Now as to the influence of the weather on the curability of the patients, we have the following data:—

Of the 8,272 patients, the months in which there were the greatest number of cures, were—

1st Series.

| | |
|---|---|
| In March ..... | 36. |
| October.... | 44. |
| June ...... | 43. |
| August .... | 42. |
| May ...... | 42. |

2d Series.

| | |
|---|---|
| In October .... | 40. |
| July ..... | 44. |
| August .... | 47. |
| June...... | 48. |

In other months the cures were less numerous.

3d Series.

| | |
|---|---|
| In September ... | 76. |
| February .... | 59. |
| May ....... | 56. |
| July ....... | 58. |
| August ..... | 60. |
| October..... | 67. |

We see by these tables, that it is at the trimestral epochs, which are marked by a change of season, that the cures have been most numerous; and especially in the month of October, in which alone there were 151 cures.

M. Desportes furnishes us with the following average relative to the cures effected.

First Series.

Of 2956 there were 881 cured—1 in 335

Second Series.

Of 2869 there were 884 cured—1 in 324

Third Series.

Of 3354 there were 962 cured—1 in 361

We are next informed that the female patients were kept longer in the hospitals than the males. The reason of this is, that the former may be more effectually and permanently cured. The men, having more means of subsistence, are of themselves generally more anxious to be discharged, when they once begin to feel themselves better. But it may be with propriety argued against this plan, that the danger of relapse must be the greater; and such, in truth, we find it to be the case.

Relapses are more frequent among the men than among the women. The following are the results of M. Desportes' report. In the first series, mentioned above, there was one relapse in every 12 men; whereas among the women there was one in every 16. In the second series, the proportion was one in every 10 among the men; and one in every 13 among the women. In the third series, the proportion was one in every 19 among the former; and among the latter one in every 22. He attributes this difference, in a certain degree at least, to the greater irregularity of life among the men, than among the women.

M. Belhomme is not quite inclined to agree with M. Desportes in this particular. He attempts to explain the difference by alluding to the difference in the cause and in the character of insanity among the two sexes. Among men, he says, it is most frequently idiopathic; whereas among the women, it is much more often merely sympathetic. Now the former, or the idiopathic insanity, is very frequently associated with lesions of the encephalon; while in the sympathetic form of the disease, there is generally no organic mischief, but only a morbidly excited state of the nervous system.

He adduces, as a proof of the correctness of these statements, that paralysis is much more frequent among insane male than female patients. This reasoning is certainly very plausible, and confirms, to a certain extent, the opinion of M. Belhomme, that idiopathic insanity affects men rather than women. We are thus furnished with a satisfactory explanation of the greater frequency of relapses among the male patients.

The following results, as to the mortality of the insane patients, are afforded us by M. Desportes' memoir. In the first series, there was one death in every six of the men, and one in every ten of the women. In the second and third series, the advantage is still more decided in favour of the women. The medium age of the deceased was, in the first series, between 49 and 50 among the men, and between 50 and 51 among the women; in the second series, the medium age among the men was 48 years, and among the women between 53 and 54; and in the third series, it was between 47 and 48 among the former, and between 50 and 51 among the latter.

As to the months in which the mortality chiefly occurred, January seems to have been the most fatal.

The observation of other physicians agrees with this statement. The severe cold of Winter appears to be very hurtful to the insane.

M. Desportes next proceeds to give an analysis of the occupations of the insane, their ages, and of the diseases from which the patients usually died.

The following table presents us with a view of the ages of the 8272 patients admitted during nine years.

| | |
|---|---|
| From 10 to 19 years of age | 626 |
| —— 20 to 29 .......... | 1568 |
| —— 30 to 39 .......... | 2024 |
| —— 40 to 49 .......... | 1683 |
| —— 50 to 59 .......... | 1051 |
| —— 60 to 69 .......... | 782 |
| —— 70 to 79 .......... | 484 |
| —— 80 to 90 .......... | 8 |
| —— 90 to 99 .......... | 2 |
| Age unknown............ | 15 |

The greatest number, therefore, was from 30 to 39 years of age.

The professions or occupations also seem to have a very marked influence on the development of insanity.

It has been remarked by many observers, that there are always a number of insane sent from the army to the public lunatic hospitals. All persons too who have been subjected to strong mental or moral excitement, as love, ambition or interest; merchants who have suffered reverses of fortune, or persons who have left off business, without betaking themselves to some active employment, are very frequently the victims of derangement.

The following table of the occupations of the patients, whom M. Desportes examined, may be interesting.

Among the male patients there were

445 day-labourers,
124 tailors,
161 shoe-makers,
101 cabinet-makers,
81 masons,
97 clerks,
62 domestic servants,
17 washermen,
1 embroiderer,
23 cooks,
1082 occupations unknown.

Among the women, there were—

924 semstresses,
397 domestic servants,
503 day-work-women,
188 washerwomen,
133 embroiderers,
88 cooks,
50 shoemakers,
339 occupations unknown.

The great frequency of insanity among females, who have been milliners and semstresses, is perhaps not to be wondered at, when we remember that so many of these poor creatures are, from their personal charms and other circumstances, more exposed than others of their sex to seduction and ultimately to destitution and poverty. The grief which follows, their anxiety and distress if they become mothers, the anguish of disappointment, are unquestionably the causes of the frequent occurrence of insanity among them.

M. Desportes remarks that insanity is more frequent among female, than among male *celibataires.* The following statement is very interesting, as it shews that the unmarried life is decidedly more exposed to the misery of mental derangement, than the "dual state," as the poet Cowper terms matrimony.

The *celibataires* of the two sexes were in the proportion of 47·16 in every hundred cases admitted into the hospitals; married persons in the proportion only of 3·55 in the 100; widowers and widows in the proportion of 13·27 in the 100; and those who had been divorced, or whose civil condition was unknown, in the proportion of 4·7.

The most frequent and powerful predisposing and exciting causes of insanity, among the patients examined by M. Desportes, will be seen by the following table:—

*Predisposing Causes.*

| | |
|---|---|
| Hereditary predisposition in | 736 cases. |
| Defect of intellectual development in . . . . | 642 |
| Premature or natural old age | 753 |

*Physical Causes.*

| | |
|---|---|
| Cerebral congestions or hæmorrhages, inducing paralysis or delirium . . . | 656 |
| Epilepsy and convulsions . | 492 |
| Efforts of menstruation, critical period of life . . | 383 |
| Consequences of parturition | 218 |
| Pregnancy . . . . . . . | 48 |
| Hysteria . . . . . . . . | 100 |
| Abuse of spirituous liquors | 414 |
| Poverty and destitution . | 109 |
| Syphilitic disease . . . | 51 |
| Misconduct and debauchery | 216 |

*Moral Causes.*

| | |
|---|---|
| Domestic distress . . . | 392 |
| Reverses of fortune . . . | 150 |
| Ambition . . . . . . . | 139 |
| Disappointed love . . . | 114 |
| Fright . . . . . . . . | 124 |
| Unknown causes . . . | 1576 |

The influence of hereditary predisposition is strongly shewn by the preceding table. The number of patients, so situated, amounts to an eleventh of the whole number of admissions, viz.

8272. The author very properly alludes to the great impropriety of marriages in certain families; but it is very doubtful whether most people will be willing to adopt his advice, "les medecins seuls peuvent etre consultés avec avantage dans ces sortes de cas."

Dr. Belhomme directs the attention of his readers particularly to the marked influence of the uterine functions, as one of the most frequent causes of insanity among females. The above table shews that not fewer than 982 cases are attributable either to the efforts of menstruation, the suppression of the catamenia, their cessation, or to pregnancy, the sequelæ of parturition, suckling, &c., or, lastly, to hysteria and other nervous affections, which are, more or less, obviously connected with the state of the uterus.

We shall now proceed to state the result of M. Desportes' inquiries as to the most frequent causes of death among insane patients.

1st Series, 1825-6-7, out of 1246 deaths,
- 253 were caused by organic disease of the encephalon,
- 395 by organic disease of the thoracic viscera,
- 445 by organic disease of the abdominal viscera,
- 108 by cachectic diseases.

2d Series, 1828-9-30, out of 1200 deaths,
- 237 were caused by diseases of the encephalon,
- 390 by diseases of the thoracic viscera,
- 393 by diseases of the abdominal viscera,
- 119 by cachectic diseases.

3d Series, 1831-2-3, out of 1408 deaths,
- 270 were caused by diseases of the encephalon,
- 468 by diseases of the thoracic viscera,
- 485 by diseases of the abdominal viscera,
- 158 by cachectic diseases.

Hence there was a total of 760 deaths from encephalic disease, 1258 from thoracic, 1322 from abdominal, and 385 from cachectic disease.

It is, however, more than probable that, in very many of the cases arranged under the thoracic and abdominal sections, there was co-existent cerebral disease—the cause of the insanity, although not of the fatal termination. The influence, which disease of other cavities has frequently on the state of the cerebral functions, is strikingly exemplified in many cases of insanity. M. Belhomme alludes to several cases in illustration of this remark, and more particularly to that of a phthisical patient, in whom the paroxysms of madness regularly alternated with the free flow of the purulent secretion, or its suppression.

---

### Comparative View of the Statistical Reports from the Hospice at Charenton, the Lunatic Asylum at Rouen, and the Tables furnished by M. Desportes from the Metropolitan Hospitals.

The report from the establishment at Charenton embraces a period of eight years; that from Rouen of ten years; and that of M. Desportes, as already stated, of nine years.

In all the three establishments the admissions were more numerous during the hot than during the cold seasons. The number of female lunatics exceeds that of the male at the Salpetriere; but is inferior at the Charenton and Rouen establishments.

In all, the age of the greatest number is from 30 to 40 years. At Charenton the number of the "modistes" and "couturiéres" is very large, as already mentioned to be the case at the Salpetriere.

In the Charenton, and also in the Rouen establishments, the number of the "femmes celibataires" is very large. At the former, the "hommes celibataires" are also very numerous. Perhaps the reason of this is, that a great many soldiers are admitted.

As to the causes of mental derangement, M. Esquirol, the physician of Charenton, states that, in one-fourth of the cases there, hereditary predisposition can be clearly traced.

In M. Desportes' report, the influence of this state is not reckoned so high.

In closing his resumé, M. Belhomme alludes to the growing frequency of insanity in France, if we may judge from the increase of admissions into the public lunatic establishments. Perhaps this is owing, partly at least, to the circumstance of families concealing the infirmity in any of their members less than formerly; but it cannot be doubted that there has been a positive increase in the malady during the last half century. We need not repeat, that the frequent political changes and public excitements have contributed not a little to this melancholy fact. It is gratifying, however, to be assured at the same time that, if the malady is increasing, the success in the treatment has of late years been decidedly greater than in former years. The improvements which have been effected in the various establishments throughout the kingdom, and the greater attention in respect both of dietetic and of therapeutic measures, have done much for this most desirable end. One of the most valuable of all the improvements is decidedly the practice of employing the insane in some regular occupation, whenever they are capable of it.

M. Belhomme says: "A very efficacious means to effect an improvement in the mental state of the insane, is to engage them in some regular occupation. Dr. Helis, physician to the asylum at Danwell, which contains between 400 and 500 inmates, has for a number of years employed his patients in the duties of the kitchen, wash-house, garden, &c. Many of the outworks of the establishments have been built by them, under the superintendance of a master."

M. Desportes has followed this example with great benefit in the Parisian hospices. By the aid of the patients alone, he has had large workshops erected, and there the inmates are constantly occupied in some light and useful occupation.—*Journal des Connaissances Medicales.*

---

### Case of confirmed Insanity cured by a sudden Fright.

A man, between 30 and 40 years of age, had been from the year 1827 to 1831 affected with an extreme degree of insanity, amounting almost to idiotcy, and alternating with periodic fits of raving madness. His condition bordered on bestiality, and none dared to approach him in his maniacal paroxysms. His case was deemed quite hopeless; and for the following two years he vegetated, so to speak, in the public lunatic house of the place. A fire having accidentally broke out near his cell, his mental powers, which had so long slumbered, suddenly were aroused; and Dr. Ollenroth, upon visiting him a few days afterwards, found him perfectly intelligent, and assiduously occupied with some domestic arrangements. He had no recollection of his former condition. All that he remembered was simply that, on the approach of the flames, he felt himself seized with an indescribable sense of anxiety, that he sprung up from his bed, and that he suddenly regained his intelligence.—*Mediz. Zeitung für Heilk. in Preussen.*

---

### Cases of Hæmorrhagic Diathesis, by Professor Kuhl, of Leipsic.

Case 1. *Hæmorrhage from a Wound.* A young man, of a pale, scrofulous, and weak temperament, wounded the skin of his thumb. The hæmorrhage continued for several days, and was so obstinate as to occasion some degree of anxiety for the patient's life. In the following Spring the skin of his forehead was cut by a tile which fell upon it. The greatest difficulty was again experienced in stopping the bleeding. Every sort of styptic, not excepting the actual cautery, was tried; but still, for several days, the wound began to bleed whenever the dressings were removed. A second and third application of the cautery were necessary at intervals of a few days, the one from the other. At length, the patient's strength being reduced to the utmost, the only security was found to be in keeping up continued pressure with the fingers. The assistants in the hospital were obliged to relieve each other at this duty for several days in succession.

CASE 2. *Spontaneous Hæmorrhage from the Surface of the Legs.*

A youth, 16 years of age, and apparently healthy in constitution, was surprised one day at finding his left stocking soaked with blood, although he had received no cut or bruise of the leg. On taking off his stocking, he observed that the blood oozed out in drops from the skin of the knee, although there was no wound. This oozing continued, without any pain, for three days, and then ceased spontaneously. But in seven weeks from this time it returned, and Professor Kuhl examined the state of the patient with minute attention. He did not exhibit any signs of scurvy, and there was nothing which indicated a dissolved state of the blood. The blood which oozed from the skin of the knee was of a bright red colour, and therefore of arterial origin. Heat increased the flow of it, and cold diminished it. Various external and internal styptics were tried, but without decided effect. Dr. Kuhl lost sight of him soon afterwards.

The Professor alludes briefly to a third case, that of a young lady, who was annoyed for a length of time with an oozing of blood from the right caruncula lachrymalis; and also to that of a child, who was brought repeatedly to death's door by spontaneous epistaxis. Sometimes upwards of a pound of blood was lost in the course of a day.—*Beitrage Zur Pract. Heilkunde.*

---

### TWO CASES IN WHICH IT IS ALLEGED THAT A LIZARD WAS VOMITED FROM THE INTESTINES ! !

*Case* 1. A woman had suffered for nearly 11 years from attacks of sharp pain in the left side of the epigastric region, of vomiting, and most distressing palpitations of the heart. The pain moved afterwards to the right side of the abdomen, and was felt lower down than formerly. The patient used always to say, that she was certain that some live body was moving up and down her bowels.

During the first few years of her illness, the paroxysms of abdominal pain returned usually every three or four months, and lasted for eight or nine days at a time. Subsequently they became more frequent. Upon first awaking in the morning, she very usually felt sick and unwell; but these symptoms passed away after breakfast. The bowels were generally much constipated. Seven years ago she became pregnant, and, although she suffered excessively from the attacks of pain during her pregnancy, she at length gave birth to a perfectly healthy child. She had tried a variety of remedies, but had not received decided benefit from any mode of treatment. On the 1st of April, 1832, she eat for supper a large quantity of acid salad (an article which almost invariably aggravated her sufferings), well peppered with strong white pepper.

On the following two nights, she experienced dreadful paroxysms of pain through the abdomen, and on the third morning, upon springing out of bed, she felt as if something living was creeping from the anus. She took hold of it, and drew it out, and, to her surprise, it was a common grey lizard (lacerta agilis) ! ! It was three inches long, and is now preserved by Dr. Bernstein, the narrator of the case. From this period the woman suffered no more from her wonted attacks of pain.

*Case* 2. A young woman had been distressed, for about two years, with periodic attacks of a most annoying feeling in the region of the stomach, as if something was moving about there; and this distress was accompanied with occasional cramp of the stomach, with nausea, and profuse flow of the saliva. The woman repeatedly described her sufferings, as if some living body was attempting to rise up her throat, and then fell down again into the stomach.

The case was regarded as one of hysteria, and treated accordingly. She then took some emetic medicines, and during one paroxysm of vomiting, she brought up a living lizard!! From this moment all her distress ceased.

The narrator of the case then proceeds to offer some remarks as to the probable mode in which the lizard came to be in this woman's stomach. He thinks it not at all improbable that it might have been developed there by spontaneous generation!! (We dare say our readers may suppose that this is a quiz on our part. Far from it; we are merely transcribing what is very coolly and seriously put down in the original report.—*Rev.*)

He is of opinion that such cases as the preceding, must be more frequent in young unmarried females than in any others; "because," says he, "the formative effort, being not engaged in normal developments, tries to compensate itself by the production of anomalous structures!!!' (This out-Herods all the *formative efforts* of transcendentalism, that we have ever heard of.)—REV.—*Wochenscrift für die gesammte Heilkunde.*

---

EMPLOYMENT OF A STREAM OF COLD WATER, IN CASES OF WOUNDS AND OTHER INJURIES.

M. Berard, surgeon to the Salpetriere, has written two lengthened articles in the Archives de Medecine, to prove the great advantages which may be derived, in some surgical cases, from keeping up a constant irrigation of cold water on the affected parts. It does not require, we think, fifty pages of print to tell what almost every surgeon knows quite well already; but if they do not contain much instructive and useful intelligence, we may be quite assured, before commencing the perusal, that we shall find in such a communication some fantastic freak, or amusing novelty.

What will the English reader think of an "irrigation d'eau froide continuée" for upwards of a fortnight, day and night, without intermission! but his suprise at this performance will be converted into mute astonishment, on being told that one of the surgeons of a hospital in Paris has practised, and recommends that a continued stream of cold water be made to pass through the wound of the abdominal parietes into the cavity of the bladder, after the high operation of lithotomy, with the view of preventing the inflammation and the infiltration of the urine, which may supervene.

After the extraction of the stone, two sounds were in one case introduced through the wound into the bladder: to the ends of these sounds were affixed flexible incompressible tubes, one of which, directed from below upwards, was plunged in a basin full of water, suspended above the bed-clothes, while the open extremity of the other hung over the edge of the bed, a basin being placed on the floor to receive the fluid which dribbled from the tube. These tubes acted as two syphons, one to introduce water into the bladder, and the other to withdraw it, as well as the secreted urine, from it. By this contrivance, the infiltration of the urine "devenait impossible," and the bladder was subjected to an " abaissement de temperature continue!!" The irrigation succeeded most admirably, the water dripping from the lower syphon as colourless as that, which flowed in by the upper one. The patient died 36 hours after the operation; but, in spite of this untoward accident, the author is quite satisfied with his experiment, because, says he, "on verra par le resultat de l'autopsie que l'eau froide avait parfaitement rempli mon attente."

Such practice requires no comment.

As it is not unfrequent that "extremes meet" in medicine, and that we hear of diseases being cured by not only the most different, but even by the most opposite and antipodal remedies, it will not perhaps be thought wonderful, that while M. Berard is lauding the good effects of cold water in the treatment of wounds, another surgeon should take the other side of the question, and descant upon the virtues of heat in the same set of cases.

Dr. Guyot has a long paper inserted in the July number of the Archives, "on the therapeutic influence of atmospheric heat." He first alludes to the recorded opinions of some surgical authors. The only one, which we shall notice, is that of Baron Larrey, who has devoted a chapter in the "Campagne d'Egypte," to illustrate the "influence salutaire du climat d'Egypte sur les plaies." The Baron mentions as a general remark, that fresh wounds healed with astonishing promptitude in that country, and that the success of severe operations was almost uniform.* In the "Campagne d'Allemagne," he has spoken of the injurious effects of the cold weather on the results of similar cases.

Dr. Guyot derived the hint from these observations of M. Larrey, to institute a suite of experiments for the purpose of investigating the effects of an elevated dry heat on the healing of wounds and sores.

He commenced his researches on rabbits. The apparatus which he used was sufficiently simple; being nothing more than a box heated with atmospheric air by means of a tube, the lower funnel end of which was placed over the flame of a lamp. In some of the experiments, the body only of the rabits was exposed to the heated air, the heads being protruded out through holes in the sides of the box, so that they thus continued to breathe the air of the room. In other experiments the animals were altogether confined in closed boxes; while in a third set, the poor devils were fastened in a row to a bench, which was perforated in the intervals between each pair, to permit the tube which conveyed the heated air from beneath to pass through. A variety of cutting and mutilating operations were performed.—Deep incisions were made in the hips; portions of the ribs were cut out, limbs were amputated, injections of cold water sometimes into the head, at other times into the belly, were performed, and so forth. The poor animals, we are informed, struggled vehemently at first, but after a short time, "leur parti etait pris," and they submitted most resignedly to the sufferings and restraints imposed on them: they gradually began to know us (the experimenters); and our presence, far from disquieting them, seemed "piquer vivement leur curiosité !!" The degree of heat, which the rabbits could bear without inconvenience, depended almost altogether on the circumstance, whether the head was immersed in the heated air or not. A temperature of 60° Cent. continued for about two hours, when the animal respired it at the same time, was sufficient to exhaust the energies of life; but when the head was kept out, the heat might be raised to 75°, and this continued for several days and even weeks continuously, without any deleterious effects. The results of some of the experiments must now be noticed. In two cases, in which simple incisions had been made through the integuments, and the animals were exposed to air heated to 60° C., union by the first intention was, we are told, effected in from four to six hours! In other two cases, although similarly treated, the wounds were not cicatrised until the third day; but during the whole time, there was no appearance of any suppuration. The results of these experiments may be contrasted with those of other experiments, in which the animals after the opcrations were confined in a temperature of only 14° C. Under these circumstances it was usually from the tenth to the fifteenth day, until cicatrisation was complete.

When the wound was accompanied with loss of substance, it was frequently observed that, under exposure to an elevated dry heat, it exsuded during the first twenty-four hours a small quantity of a viscous serosity; that on the second day its surface was covered with a reddish coloured varnish; that on the third a deposit of "une matiere blanche solide" (coagulable lymph?) was observed at various points, and that this gradually extended over the whole surface, the edges of the wound contracting at the same time, until perfect cicatri-

* The results of M. Clot-bey's surgical practice in the Hospital of Cairo and Abouzabel confirm the accuracy of Larrey's Observations.

zation was effected, at an interval from the operation of from ten to twelve days.

In most of the cases so treated, it was particularly observed that the wounds were free from inflammation, and that the suppuration was greatly less profuse, than it usually is under reduced temperatures.

All the amputations appear to have proved fatal; and, as to the results of other operations, we may avail ourselves of the author's own avowal; " dans un grand nombre de cas, nous n'avons pu saisir de difference dans l'action des instrumens chauds et froids."—We pass now to a brief summary of the results of Dr. Guyot's trials of his remedy on the human subject.

M. Breschet, surgeon of the large hospital, the Hôtel Dieu at Paris, afforded him the requisite opportunity. The apparatus used was a very simple one, and quite similar to that employed at vapour bath establishments, to expose only one part of the body to the operation of steam, or of heated air.

The first case was that of a young postillion, who had laboured for nearly four years under an ulcer on the upper and inner part of the leg, the result of an old compound fracture. Six months after the receipt of the accident a considerable portion of the tibia had been extracted, and, ever since that date, the wound had remained open. The poor fellow had come to Paris to submit to the amputation of his limb. M. Breschet, whose patient he was, placed him under the care of Dr. Guyot. The ulcer was about three inches in length, and two broad, and there was another sore of smaller dimensions. The purulent discharge was scanty. After two hours application of air heated to 45° C. the edges of the wound exhibited the appearance of having been dried: after a five hours' application, the sores were found to be covered with a thin crust, under which a quantity of serosity had been deposited.

On the morrow, the two sores were covered with a dry crust, without any fluid underneath. The general health of the patient at this time began to be much disturbed; and this he attributed to the effects of the heat. It was discontinued, and forthwith the purulent discharge increased in quantity. The apparatus had been applied for only thirty-six hours, and already it had changed " completement l'etat de la plaie, et la marche de son travail."

The patient subsequently underwent amputation, and died soon after the operation.

The second case occurred in an old man, on whose left inner ancle there had been a varicose ulcer during the long space of twenty-five years. Like most ulcers of this sort it occasionally almost quite healed up, and then it broke out again. It had been open at the date of this report for upwards of eight months. The edges were swollen and exceedingly painful; the discharge was sanious and of a greyish colour.

The limb having been exposed for several hours to an atmosphere heated to 45° C., the inflammatory irritation was greatly reduced, and the wound had exsuded a quantity of serosity. On the following day, the ulcer was covered with a crust, under which was a thick inodorous pus. For ten days the air-bath was continued without intermission; only at times the lamps had gone out, and the temperature of the bath had thereby become reduced. At these times, the patient had uniformly complained of the pain having returned in the sore. With occasional interruptions, the treatment was persevered in from the 27th of November, the first day of the application of the apparatus, to the 3rd of the following January, by which time the sore was completely cicatrised. A month after the patient's dismissal from the hospital, the limb remained quite well. Two other cases are recorded by Dr. Guyot, in illustration of the good effects of the continued application of heated air to chronic ulcers.

The memoir, from which the preceding extracts have been taken, was laid before the Academy of Sciences, and M. Roux was appointed to report on the practice recommended. He entrusted some of his patients in the La Charité to M. Guyot.

A chronic ulcer on the ancle of an old man was cured in the course of 14 days; a white-swelling of the knee-joint was, we are told, greatly relieved; and an old fistulous sore on the heel speedily healed up, by the foot being kept constantly in an atmosphere heated to 40° C. But these data are quite insufficient to warrant any deductions favourable to M. Guyot's proposal; and we ask him why he has not published M. Roux's report. The verdict of such a man as M. Roux, one of the first surgeons in Paris, must have deserved recording; and, if it had been favourable, we doubt not it would not have been kept back.

The heated air-bath is no doubt a valuable remedy in some diseases. Certain ulcers may possibly be benefited by it; affections of the joints are certainly much relieved by its application; so are some local cutaneous eruptions, rheumatic and neuralgic pains, &c.—*Archives Generales.*

---

### Reduction of an Old Dislocation of the Shoulder — Death the same Day—Appearances of the Joint on Dissection — Remarks on M. Lisfranc's Character as a Surgeon.

The patient was a robust plethoric man, 40 years of age. The dislocation, upwards and inwards, had lasted for four months. He was bled previous to the attempts at reduction, and this was effected without the aid of pulleys by several assistants, under the direction of M. Lisfranc, who, while the extension was making, lifted up the head of the bone by means of a towel passed under the patient's arm and over his own neck. Three hours after the reduction, the patient suddenly died from an apoplectic attack.

On the dissection of the joint, an ecchymosis was found to extend from the lower edge of the pectoralis major muscle to the middle of the neck, and from the sternal extremity of the clavicle to the subscapular and infraspinal fossæ. The effused blood permeated all the tissues from the skin to the subjacent pleura. The subclavian and axillary artery and vein, and the plexus of nerves, were not injured. When the deltoid and pectoralis muscles were lifted up, the head of the bone was found under the acromio-coracoid arch. It evidently was more prominent than in the natural state of the parts, and this projection seemed to be caused by something interposed between the round head of the humerus and the glenoid cavity. It was enveloped in a dense fibrous tissue, which had all the appearance of the ordinary capsular ligament.

On continuing the dissection, it was evident that this enveloping tissue was a new formation, and that the puckered and altered remains of the original capsule were interposed between the head of the bone and the glenoid cavity of the scapula.

The heart was found hypertrophied, and the vessels of the brain and of its membranes excessively gorged with blood, although no traces of sanguineous effusion were discovered.—*La Lançette Française.*

We do not charge M. Lisfranc with any blame for the unfortunate issue of the preceding case—far from it. The same accident might have occurred in the practice of a much more cautious surgeon than M. Lisfranc is. But there has been an accusation of wilful misrepresentation of truth lately brought against him by one of his pupils; and this we feel ourselves bound to notice. In the course of last year, M. Lisfranc communicated to the Academy of Medicine a list of 84 cases, in which he alleged that he had successfully amputated the cervix uteri. This, indeed, is a most startling and most unexpected announcement; and one which, even in the present day of medical wonders, makes a large demand upon our credulity. But what will our readers say, when they are informed that Dr. Pauly, who tells us that he was "chargé de rechercher les materiaux" of the memoir presented by his former master to the Academy, has assured the public in his recent work, "Maladies de l'Uterus," that the statements of M. Lisfranc are not true; that, of all surgical operations, the amputation of the neck of the uterus is one of the most murderous (meurtrieres); and that every case, in which it was performed, "lui offrait une victime," the poor patients, when dismissed from the hospital, being told by M. Lisfranc: "Allez, vous etes gueries; vos douleurs se desesperant à la longue."

We admit that the prevailing tone of Dr. Pauly's work indicates a personal hostility to M. Lisfranc; and we are bound to await the latter's reply. But we must confess that the perusal of the lectures "du grand chirurgien de l'Hôpital de la Pitié" has not given us a favourable impression of his practical talents. He is rash—and rashness is one of the very worst attributes of a medical man.

In a late number of the Gazette Medicale, we read the history of a case, where a woman died of violent metritis, which supervened on the very same day on which M. Lisfranc had made a tedious and painful examination of the uterus for suspected polypus.

His vaunted discovery of the great utility of muriates of lime and barytes, in white swellings of the joints, is not likely to obtain much credit among British surgeons.—*Rev.*

---

### CURIOUS CASE OF SIMULTANEOUS DISLOCATION OF BOTH THIGHS.

A sailor was sitting astride a plank, when a wave suddenly forced him up against a cross beam, which struck his back violently, while the plank was still between his legs. The poor fellow was lying on his back, when Dr. Sinogowitz was summoned to his assistance. Both limbs were quite motionless, and evidently much deformed from their natural figure. The thighs were separated the one from the other, and could not be approximated; the trochanters were much lower and less prominent than usual, and the muscles of the hips over them were in a state of extreme tension. The body was bent immovably forwards and downwards upon the thighs; the knees were moderately flexed, and the toes were not turned either inwards or outwards. The diagnosis, therefore, was that the heads of both of the thigh bones were dislocated downwards and inwards. The reduction was effected in the following manner. The pelvis being secured by two assistants, the surgeon took his place between the limbs of the patient, and having put a towel round the right thigh above the knee, he passed the noose of it over his own neck. Extension was then made by means of a towel made fast above the ancle, and inclined a little to the left side, and while this was steadily continued, Dr. S. lifted the head of the bone, and directed it upwards and somewhat outwards, by raising and stretching out his head with all his power. It slipped into the socket without any noise. The left limb was then reduced in nearly a similar manner. The mobility of the limbs was almost immediately restored, at least in the horizontal position; but several months elapsed before the patient could walk with any degree of ease. The tediousness of the recovery was owing in a very great measure to the severe injury of the lumbar vertebræ, which he sustained at the time of the accident. For three weeks, the sphincters of the bladder and rectum were quite paralysed. —*Preussische Medicin. Zeitung.*

---

### VARIETY OF CLUB FOOT TREATED BY THE DIVISION OF THE TENDO-ACHILLIS.

A man, 46 years of age, had been affected from his childhood with that variety of club-foot which has been denominated "horse-foot," (pied equin), and which consists in the person walking on the point of the foot.

The infirmity was not congenital, but had been the result of a wound of the heel from the bite of a dog, when the patient was six years of age. From inattention the patient had ever since that time rested on the point of the affected foot in walking, and the consequence of this proved to be a permanent and unyielding extension, the heel being drawn up by the contraction of the tendo-achillis. The ancle-joint was quite sound; the calf of the leg was somewhat smaller than that of the opposite limb.

The remarkable success obtained by M. Stromeyer, of Hanover, and by M. Duval, at Paris, from dividing the tendo-Achillis in such cases, induced the attendant surgeon to propose the operation in the present instance. M. Roux was consulted, and approved of the practice suggested. A long straight bistoury was passed behind the tendon, at about six finger-breadths above the external malleolus, in a direction parallel to that of the tendon. The blade of the instrument shaved, as it were, the posterior face of the fibrous bundle; but its point was not made to penetrate through the skin of the opposite (the inner) side. This being removed, a probe-pointed bistoury, with a very convex blade, was introduced along the wound, so as to divide across all the fibres of the tendo-achillis. The foot could then be brought to its proper position, the heel being drawn down into a line with the toes; but it was thought advisable to permit the wound to heal, and a deposition of " tissue inodulaire" to take place between the divided ends of the tendon, before the permanent extension was resorted to. The instrument employed was somewhat like the boot of J. L. Petit, only that it acted in an opposite direction—of keeping the foot constantly bent upon the leg. It consisted of a padded and laced shoe, to which were attached two lateral splints or rods, which extended up to the knee, where they were secured in their places by leather straps. A leather strap extended from the point of the shoe to a circular piece of metal, which passed round, and was made fast to the splints about the middle of the leg. By shortening and lengthening this strap, the toes could be readily elevated or depressed.

We are told that the patient was very restless and intractable. However, by the fifteenth day after the operation (the date of the report), the foot had resumed " une grande partie de ses fonctions normales."—*Gazette des Hôpitaux.*

The practice of reporting cases, and this too as being quite successful, before the result is known, is very common among our French brethren. On the present occasion, the reporter " engage les chirurgiens à imiter la pratique qu'il vient de decrire," although, in the paragraph immediately preceding, he had expressed his doubts as to the ultimate success of the case.

The following abstract of four cases, which we find narrated by Stromeyer himself in a late number of Rust's Magazine, deserves notice. The first was that of a boy, seven years old, who had been born club-footed in both limbs. The right foot was most deformed. The tendo-Achillis was divided. The wound healed speedily by the first intention; but the extension was not commenced till eight days after the operation. Whether the connecting cellular tissue had by this time become too unyielding to permit the foot being brought into a natural position, or there was some other unfavourable condition present, we are not informed; but it appears that the patient was not at all benefited by the operation which had been performed, and the parents would not consent to a second one being performed, as proposed by Stromeyer.

The second case was more successful. The disease was not congenital, but had come on, without any very evident cause, when the boy, now 13 years of age, was in his fourth year. The twisting of the foot was even worse than in the preceding case; and in addition to the general deformity, the big toe was permanently contracted downwards and inwards, in consequence apparently of the flexion of the tendon of the flexor longus pollicis. Dr. Stromeyer was of opinion, that before attempting to remedy the greater evil, it was advisable to rectify the deformity of the toe. The tendon of the flexor longus was therefore divided, and three days afterwards the toe was extended, and kept in that position for a week. The more important operation was then performed. Extension was commenced on the fifth day. In the course of ten days, the foot had been brought to an angle of 70° with the leg. Four weeks after the operation, the extension-apparatus was exchanged for the mechanical boot which has been contrived by Stromeyer for the purpose, and the patient allowed to walk out. Half a year afterwards, he could move about with the greatest ease, and with the exception of a slight turning-in of the point, the foot had regained its normal shape and mobility.

In the third case, that of a boy nine years old, the disease was congenital. The toes and the metatarsus of the right foot were bent strongly downwards, so that the dorsum of the foot formed quite a convex line with the leg, and the great toe was drawn upwards in a strange manner towards the foot, so that the only point of support, when the patient stood or walked, was the metatarsal joint of this toe. The tendo-Achillis was divided; and on the fifth day afterwards the extension apparatus was put on.

As the point of the foot was still drawn considerably inwards by the contraction of the tendon of the flexor longus pollicis, this was divided, and extension afterwards kept up.

In about ten weeks after the date of the first operation, the form and mobility of the foot left nothing to wish for; and the boy could move about with the greatest facility.

The fourth case occurred in a youth 19 years of age. The foot was turned inwards, so that the point of support in standing was on the metatarsal bone of the small toe. The result of the operation and of the subsequent treatment was so successful, that the deformity was quite removed (das entstellende uebel war ganz gehoben.)

---

### Wry-neck—Section of the Sterno-Mastoid Muscle.

The patient, 53 years of age, stated that seven years ago, while carrying a heavy weight on his back, he suddenly felt a sharp pain on one side of the neck, and that this pain lasted for the following fortnight or three weeks; that some time after all uneasiness had ceased, he began to experience, chiefly at night, a stiffness of the neck, and a tendency to incline it to the left side; that on awaking one morning, he found that he had a very painful wry-neck; that this attack however speedily abated; but that ever afterwards the stiffness of the neck and its turning to the left side were much more troublesome than they had been before, and that these symptoms soon increased so much, that he was forced to resort to the expedient of steadying his head, when engaged at his work, by fixing a packthread to his front teeth, and securing it to one of his thighs! This expedient however proved insufficient; and in the course of ten months, the malady had increased so much that he was forced to discontinue altogether his employment as a shoemaker. The left sterno-mastoid had become larger and thicker than its fellow. A variety of remedies, including blisters, acupuncturation, electricity, &c. had been tried without avail. M. Amussat therefore advised him to submit to the division of the affected muscle. Pinching up the skin, about an inch above the insertion of the muscle into the extremity of the clavicle, he divided the fold with a sweep of the bistoury; he then severed the muscle, layer of fibre after layer, permitting one to retract before dividing the other, until the entire substance was fairly cut through, with the exception of a few of the outer, or clavicular fibres. A few arteries sprung; but the hæmorrhage from these was easily arrested, by twisting their bleeding extremities. The wound was then dressed with simple cerate. The wryness of the neck was unexpectedly quite as great after, as it had been before, the operation, and continued to be so, for at least three weeks. As the cicatrisation of the wound advanced, the deformity was observed to decrease; and, by the end of the sixth week after the operation, when the wound was quite healed, the normal position of the neck was perfectly recovered.

The most interesting features of the present case are, first, the long continuance of the malady; it had existed for seven years; secondly, the cure obtained without the use of any apparatus, either during or after the section of the muscle; thirdly, the permanence of the cure—it is now upwards of a twelvemonth since the date of the operation; and lastly, the proof which is thus established of the efficacy of the treatment recommended by the older surgeons, and which of late years had fallen into unmerited desuetude.

The point, at which M. Amussat divided the muscle, was that usually indicated in surgical works. M. Malgaigne has, in his Manuel Operatoire, suggested that the division should be made higher up, with the view of more effectually avoiding the large bloodvessels in the neighbourhood, and also because the muscle is less bulky. With respect to the former of these motives, it is founded on an anatomical mistake; for M. Amussat shews that the sterno-mastoid is as close to the bloodvessels higher up in the neck, as it is lower down; and moreover that at this latter point, the omo-hyoideus being interposed between the muscle and the vessels, secures the latter in some degree from the risk of injury.—*Gazette Medicale.*

---

### Successful Case of Amputation at the Hip-joint.

A countrywoman, 25 years of age, was admitted in August 1832, into the surgical hospital at Erlangen. From the age of 12 to 15 years she had had a fistulous abscess, situated immediately above the internal condyle of the left thigh. When this abscess healed, her health was remarkably good, and continued so until about a twelvemonth previous to her admission into the hospital. At that time the whole of the left lower extremity became swollen and painful. Two abscesses formed above the condyles, and, when they broke, discharged a large quantity of pus. The patient became hectic, amputation was proposed, and she at length submitted to it. The knee was permanently bent; it was much enlarged, and extremely tender on pressure.

On introducing a probe along the fistulæ, the bone was found to be carious and necrosed. The limb was amputated in the upper third of its extent. On sawing through the bone, it was discovered that it was unsound. On detaching the muscles from it for two or three inches, M. Jaeger discovered that the trochanters were affected with caries. He now determined to disarticulate the head of the femur. This was done without much difficulty.—The acetabulum fortunately was quite healthy. Very little blood was lost in this second operation. The quantity of soft parts was much greater than was necessary to form a good stump; but M. Jaeger was unwilling to subject his patient to more suffering, as already she was in a weak and fainting state. He therefore united the wound with a few stitches, leaving its lower angle free, to permit the issue of any discharges. The stump, enveloped in linen wet with cold lotion, was laid upon a cushion of bran, and no bandages were applied.

On the third day after the operation, three of the stitches and also the strips of plaster were removed, in consequence of the lips of the wound having become somewhat swollen and painful, and dressings of simple cerate were applied, and retained in their place by a few turns of a roller. Light cordials and nourishing broth were allowed, in small quantities at a time, as the vital powers were rather low and apt to flag. The upper angle of the wound having assumed somewhat of a livid hue, it was frequently bathed with aromatic fomentations, sharpened a little with pyroligneous acid. Under the application of these, continued for ten or twelve days, this disagreeable appearance gradually subsided, and at the same time the character of the purulent discharge was much improved.

By the end of the fourth week the wound was nearly cicatrised; the general health of the patient improved rapidly; and menstruation, which had ceased for upwards of a twelvemonth, returned on the seventh week after the operation.

The state of the amputated limb was as follows.

The subcutaneous cellular tissue around the knee was thickened to a very considerable extent. All the parts of the joint were carious; the femur was thickened and uneven; and on its posterior surface were three openings, through which might be felt the sequestrous bone. On examining the bone, where it was sawn through at first, it was found to be internally carious; and this unhealthy state might be traced upwards as high as the two trochanters. The head and neck of the femur were internally soft, and very vascular.

Professor Jaeger, in discoursing on the preceding case, attributed its successful termination in part to the simple manner in which the bone was disarticulated. He is inclined to recommend surgeons to consider more attentively than they have done hitherto the plan which he followed—viz. to perform the circular operation first, and then to extirpate the head of the bone from its socket.

The state of the patient's health too was favorable to the cure. Naturally of a sound constitution, there was precisely that degree of feebleness, which keeps down inflammatory reaction, and which thus is decidedly favorable to recovery after great surgical operations. The simple mode of dressing the wound at first with cold fomentations, and the abstaining from all compression with bandages and so forth, is strongly recommended.—*Schmidt's Jahrbucher.*

---

### CASES OF AMPUTATION.

1. *Amputation at the Hip-joint.* A soldier 24 years of age, belonging to the French troops at Algiers, was struck in the left thigh, by a ball which had entered four inches above the knee, and had shattered the os femoris into several pieces. The fracture was in the lower third of the bone. Amputation was proposed, but not acceded to. Several fragments of bone were extracted, and the limb being properly secured, the poor fellow was sent down from the Atlas, where the action had taken place, to a hospital at the foot of the mountain. M. Baudens, the surgeon in chief of the forces, did not again see him for six days. The bandages had by this time become hard and thickened with blood and matter; but as there was not much tumefaction of the limb, they were not removed (was this good surgery?—*Rev.*), and the patient, although suffering from fever and great constitutional irritation was transported in a litter to the General Hospital at Algiers. When the limb was examined, it was found that "les fusées purulentes s'etendent jusqu' au dela du grand trochanter, et par la compression le pus s'echappe à flots!!" The poor fellow himself, now wasted with hectic, implored M. B. to amputate if there was any chance of saving his life. Some of the surgeons in consultation dissuaded this step, as the purulent sinus extended above and around the trochanter major, and as moreover the strength of the patient was greatly exhausted by the protracted suffering; but, as the flesh on the fore and inner part of the limb was quite sound, M. Baudens was of opinion that a sufficiently large flap might be obtained from it to cover the joint. The operation was performed on the fourteenth day after the accident. The inguinal artery was compressed over the pelvis, and the limb removed in forty seconds. The flaps formed were rather anterior and posterior than internal and external, as recommended by most surgical authorities. Not more than four or five ounces of blood were lost. The main artery was tied, and two smaller ones were twisted (tordues). The patient having fainted, a quarter of an hour was permitted to elapse, and, as no other vessels required securing, the lips of the flaps were then brought together and kept in their places by means of six sutures. Light nourishing broth, and antispasmodic medicines were exhibited at short intervals. For six days the patient remained very low. On the seventh the dressings were removed for the first time, and the wound was found to have cicatrised along its whole length, except at the two extremities. The ligature (we suppose, the chief one) came away on the fifteenth day. By the end of the fifth week, he was able to move about on crutches, and the wound was quite healed in the course of another week.

2. *Amputation at the Knee-joint.*

A soldier in the skirmish at Tafna, 26th January, 1836, received in his right knee a ball, which, after shattering the patella, lodged in the inner condyle of the os femoris.

M. Baudens having attentively examined the wound, judged that the case was one well suited to the performance of amputation in the joint itself. He commenced the first incision at the crista of the fibia, two inches below the patella ligament, and carried it round on each side to a point, an inch and a half below the centre of the popliteal space. The integuments in front were then carefully dissected upwards as high as the upper edge of the patella; the joint laid completely open all round by dividing the capsular and other ligaments, and, while an assistant compressed the popliteal vessels between his fingers, an ample muscular flap was made from the muscles on the back of the leg to cover the end of the femur. It was found necessary to remove portions of both condyles, as they had been fractured, and the ball still lodged nearly an inch and a half deep in the substance of the bone. Five ligatures were applied, and the wound was secured by three sutures. The healing process was considerably retarded by the oozing of blood from the wound; but eventually a good serviceable stump was obtained.

3. *Amputation in the Knee-joint. Line of the incision below one of the Gun-shot Wounds.*

A young soldier in the action at the foot of the Atlas, on the 1st of April, 1836, was wounded by a musket ball, which entered immediately beneath the lower edge of the left patella, and came out in the centre of the ham. The head of the tibia was broken into several fragments.

The skin of the leg being drawn strongly upwards, M. Baudens made a circular cut through the integuments. The line of this incision was kept about five finger-breadths below the inferior edge of the patella, and three inches below the condyles of the femur posteriorly. The integuments were then dissected upwards, all round, except behind; the capsular and other ligaments divided, and, while the popliteal artery was compressed by an assistant, the posterior flap was formed by one stroke of the knife. Only one artery required ligature. The lips of the wound were held together by four stitches of the interrupted suture. The healing was speedy and perfect.

M. Baudens says that the circumstance of the gun-shot wound in front being above the line of the anterior flap was rather favorable than otherwise to the cicatrisation. It afforded a convenient outlet to any pus which formed. The line of the cicatrix on the face of the stump was situated well back in the popliteal space, so that it quite escaped pressure when the patient used a wooden leg.—*Lançette Française, Juillet*, 1836.

The operation of amputating in the knee-joint is never likely, we should suppose, to be generally or even frequently adopted. It possesses no advantages, unless perhaps that of saving two or three inches in the length of the stump; and it is certainly liable to numerous and very serious objections. One of the Paris hospital surgeons, (M. Velpeau, we think) has very recently been *experimenting* on this subject, and the result of his trials in three or four cases has been that every patient died.

---

Radical Cure of Varicose Veins and of Herniæ by Acupunctuation.

M. Bonnet, chief surgeon of the Hôtel

Dieu at Lyons, informs us that he has treated eleven cases of varicose veins by introducing pins through their cavities, and allowing them to remain there for some time. Nine of these cases were cured. He has applied the same treatment to herniary sacs; and the following is a short account of his method, and of the success which he has obtained from it. He passes three or four pins through the herniary coverings close to the inguinal ring, and in order that they may exert a certain degree of compression, as well as of irritation, on the sac, he twists upwards their points and heads, so as to give them a circular direction.

Caution is necessary not to injure the spermatic cord. The inflammation and pain commenced usually on the third or fourth day after the operation, and the pins were removed a few days afterwards. M. Bonnet has treated four cases of inguinal herniæ by acupuncturation. In two of these, the herniæ were small, and three weeks sufficed for the cure. The third was more troublesome. It occurred in an old man 67 years of age; and in him the hernia descended to the bottom of the scrotum, and was with difficulty kept up by a truss. Six needles were used. After a month's treatment, this patient could walk about, without any tendency of the viscera to descend. In the fourth case, the hernia was of 30 years' standing; no truss could keep it up; the inguinal aperture was large enough to admit " l'introduction de cinq doigts reunis," and the tumor descended a considerable way down the thigh. Five weeks were necessary for the cure. We are assured that all these patients could cough and walk about freely without any escape of the bowels, and that the inguinal ring was so plugged up, that it could no longer be distinctly recognized.—*Bulletin de Therapeutique.*

The proposal of M. Bonnet is certainly more worthy of notice, and promises better success than that of M. Gerdy, which we noticed in the Foreign Periscope of a recent number.

---

### SPONTANEOUS GANGRENE OF THE LEG —AMPUTATION BEFORE THE LINE OF SEPARATION WAS ESTABLISHED.

A woman, 40 years of age, experienced quite suddenly an icy coldness and very painful cramps in the left leg, from the toes to the knee. She fell down in a state of almost complete insensibility. The surface of the body was cyanosed, the action of the heart was tumultuous, no pulsation could be felt at the wrist, and there was dyspnœa, amounting to almost complete orthopnœa. The affected leg was of a marbly white colour; the toes were contracted convulsively, and the calf of the leg was excessively painful, the pains being much aggravated by any movement. This woman had been labouring under disease of the heart for several years. On the morrow the affected limb became of a livid red colour, and swollen as far as the knee. Phlyctenæ began to be formed at several points, and the patient complained of a burning heat in the limb. Two days afterwards there was much feverish disturbance, accompanied with some mental confusion; the limb was still very painful; it was black from the toes to the middle of the calf, of a livid red hue thence to the knee, and covered with several large phlyctenæ. An inflammatory redness affected the outer and back part of the thigh, as high up as eight inches above the knee.

M. Amussat amputated the limb at the lower part of the thigh; four vessels were tied. The wound healed by the first intention. On dissecting the amputated limb, a coagulum was found in the popliteal artery, adhering to its internal membrane, and extending for the distance of some lines into the anterior tibial. The popliteal vein also was plugged up in the same way as the artery: its walls were much thickened, and seemed more like those of an artery than of a vein. The sciatic nerve at the same point was found to be swollen, and of a dark hue.

